# Octylamine-Modified Cellulose Nanocrystal-Enhanced Stabilization of Pickering Emulsions for Self-Healing Composite Coatings

**DOI:** 10.1021/acsami.2c01324

**Published:** 2022-03-07

**Authors:** Guofan Xu, Rinat Nigmatullin, Todor T. Koev, Yaroslav Z. Khimyak, Ian. P. Bond, Stephen J. Eichhorn

**Affiliations:** † Bristol Composites Institute, School of Civil, Aerospace and Mechanical Engineering, University of Bristol, University Walk, Bristol, BS8 1TR, U.K.; ‡ School of Pharmacy, University of East Anglia, Norwich Research Park, Norwich, NR4 7TJ, U.K.

**Keywords:** cellulose nanocrystals, octylamine, linseed oil, Pickering emulsion, self-healing, coatings

## Abstract

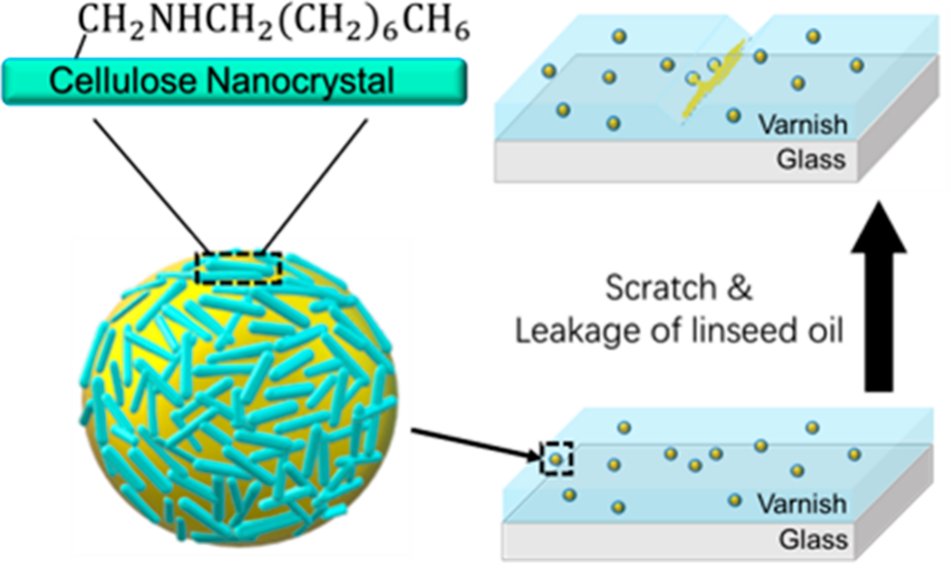

Linseed oil-in-water Pickering emulsions are stabilized by both sulfated CNCs (sCNCs) and octylamine-modified CNCs (oCNCs). oCNCs with hydrophobic moieties grafted on the surfaces of otherwise intact nanocrystals provided emulsions exhibiting stronger resistance to creaming of oil droplets, compared with unmodified sCNCs. sCNCs were not able to completely stabilize linseed oil in water at low CNC concentrations while oCNCs provided emulsions with no unemulsified oil residue at the same concentrations. Oil droplets in oCNC emulsions were smaller than those in samples stabilized by sCNCs, corresponding with an increased hydrophobicity of oCNCs. Cryo-SEM imaging of stabilized droplets demonstrated the formation of a CNC network at the oil–water interface, protecting the oil droplets from coalescence even after compaction under centrifugal force. These oil droplets, protected by a stabilized CNC network, were dispersed in a water-based commercial varnish, to generate a composite coating. Scratches made on these coatings self-healed as a result of the reaction of the linseed oil bled from the damaged droplets with oxygen. The leakage and drying of the linseed oil at the location of the scratches happened without intervention and was accelerated by the application of heat.

## Introduction

Emulsions are mixtures of multiple immiscible liquids. In an emulsion, one liquid is separated into small droplets and dispersed in another continuous liquid phase with stabilizers at the interfaces. In 1907, Pickering^1^ showed that the coalescence of oil droplets in water was prevented when they were encapsulated with paraffin-insoluble solid particles. Since this pioneering work, emulsions stabilized by solid particles are generally called “Pickering emulsions”. It has been demonstrated that the adsorption of particles with contact angles close to 90° (partial wetting condition) at the oil–water interface can be considered irreversible, resulting in very stable Pickering emulsions.^2,3^ Particles with a hydrophilic surface form oil in water (O/W) emulsions while those with a hydrophobic surface form water in oil (W/O) emulsions.^3^ The average droplet size and emulsion stability have been demonstrated to vary with the size^4^ and surface wettability of the particles.^3^

Various types of particles satisfying the partial wetting condition have been used to stabilize different kinds of oil–water interfaces. Both organic and inorganic particles like latexes,^4−6^ silica,^7,8^ layered silicates such as bentonite and laponite clays,^9,10^ carbon nanotubes,^11^ graphene oxide,^12,13^ and magnetic particles^14,15^ have been reported to assist in forming stable Pickering emulsions. Biocompatible and biodegradable emulsions have also been investigated using block copolymer micelles,^16^ spore particles,^17^ protein particles,^18,19^ and cellulose nanomaterials^5,20−24^ as stabilizers. The particle wettability in oil and water can be tuned by the adsorption of surfactants^7,25,26^ or by chemical surface modification.^5,21,27^ Chemical modification is thought to be more reliable than adsorption as a result of chemical bonds between the particle’s surface and the grafted molecules, while adsorption can be disrupted by solvent conditions.^2^

Cellulose is a biopolymer that is readily functionalized, abundant in biomass, and has many industrial applications. Cellulose nanomaterials (CNMs), with highly ordered crystalline structures, nanoscale sizes, and high aspect ratios, can be extracted from biomass via either mechanical and/or chemical processing.^28^ CNMs are inherently hydrophilic, but their hydrophilicity can be moderated by surface modification to maintain contact angles at interfaces of ∼90° for stable adsorption.^29^ CNMs have been used to stabilize oil phase polymers in water and form microscale oil droplets, providing stable Pickering emulsions.^20−23,30−34^ These resulting Pickering emulsions can then be used to create microcapsules or microparticles by interfacial chemical reactions, by in situ polymerization, or by simple drying.^5,20,22,24,30^ Kolanowski et al.^24^ used methyl cellulose (MC) and hydroxypropyl methyl cellulose (HPMC), soluble forms of derivatized cellulose, combined with low-viscosity maltodextrin and soy lecithin as additional emulsifiers to prepare fish oil emulsions in water. These emulsions were then spray-dried to form fish oil microcapsules with MC or HPMC walls.^24^ The oil encapsulating level reached 98.5% for a sample with 400.0 g/kg fish oil, the highest among all samples and 10% higher than common fish oil powders.^24^ However, a coemulsifier of soy lecithin was used because MC alone cannot ensure the stability of fish oil droplets during the spray-drying process. Kalashinikova et al.^23^ used hydrochloric acid hydrolyzed bacterial cellulose nanocrystals (BCNs) as the sole stabilizer, creating a hexadecane in water Pickering emulsion. The emulsion stability was tested by centrifugation and quantification of the cellulose amount released in the aqueous subphase after centrifugation was obtained.^23^ The emulsion was demonstrated to be stable, with no droplet size variation after centrifugation, and long-time storage at low temperature or when being kept at 80 °C for 2 h.^23^ No BCNs were found in the separate aqueous phase after centrifugation, proving the irreversibility of the adsorption process.^23^ The percentage of encapsulated hexadecane increased to a plateau at ∼70% with a BCN concentration higher than 1.4 g/L. Such oil phase content is close to the “theoretical close packing condition of 0.74 for monodispersed spheres”.^23^ The ability of cellulose nanocrystals (CNCs) to emulsify paraffin droplets in water was tested by Han et al.^20^ and compared with that for samples with additional cationic surfactants. With ζ potentials ranging from −52.1 to −30.5 mV, under different pH values, CNCs were found to stabilize O/W emulsions after sonification but “creamed” because of intercellulose interactions and the low density of CNCs covering the oil droplets. Creaming is a migration of the dispersed phase in an emulsion under the influence of buoyancy. After 12 h, the Pickering emulsion, without the presence of additional surfactants, separated into two layers, with lighter paraffin droplets close packing with each other in the upper layer and water being excluded in the lower layer.^20^ This creaming phenomenon did not occur in the samples with additional surfactants and the average droplet size decreased owing to their presence. Han et al.,^20^ therefore, stated that CNCs alone were not suitable for emulsifying paraffin in water-based Pickering emulsions.

Covalent surface modifications have been used to solve the flocculation problem of nanocellulose-stabilized Pickering emulsions, which was caused by the abundant hydroxyl groups on unmodified cellulose structures.^21,31−33^ Acetylation has been used to chemically modify cellulose nanofibers (CNF), reducing their surface energy and hydrophilicity by partially replacing the hydroxyl groups.^21^ Acetylated CNFs (AcCNFs) were found to provide a higher paraffin encapsulation efficiency since they are found to be more compatible with this liquid.^21^ The AcCNFs successfully emulsified paraffin in water at 80 °C, and the droplets solidified into microparticles with AcCNF networks adsorbed on the particle surface after cooling.^21^ Thermoresponsive poly(NIPAM) brushes were grafted on CNCs by Zoppe et al. and the heptane-in-water emulsions using these materials were reported to be stable for 4 months.^31^ Tang et al. prepared pH and temperature sensitive heptane-in-water and toluene-in-water emulsion systems using polyelectrolyte, poly[2-(dimethylamino)ethyl methacrylate] (PDMAEMA) modified CNCs.^32^ These emulsions responded to pH owing to a changing chain conformation of PDMAEMA.^32^ Chen et al. increased the surface hydrophobicity of CNCs by modifying CNCs with octenyl succinic anhydride (OSA) and obtained gel-like Pickering emulsion systems.^33^

Noncovalent functionalization of CNCs can also be used to achieve higher surface activity through the adsorption of polymers.^22,34^ Kedzior et al.^22^ bound MC on sulfuric acid hydrolyzed CNCs and used these modified materials to make methyl methacrylate (MMA) in water emulsions, which were then made into a PMMA particle suspension through in situ polymerization. The MCs were reversibly adsorbed onto the CNC surface upon mixing, and both MC and MC-coated CNC emulsified MMA in water. Therefore, a double morphology appeared in the MMA capsules and, subsequently, the polymerized PMMA particles. Cationic surfactants didecyldimethylammonium bromide (DMAB) and cetyltrimethylammonium bromide (CTAB) have both been adsorbed onto the anionic CNC surface.^34^ However, the surfactants CTAM and DMAB dominated the emulsification process at high surfactant concentrations, with only small amounts of CNCs being adsorbed to the oil–water interfaces.^34^

Nigmatullin et al.^35,36^ modified sulfated CNCs (sCNCs) with hydrophobic alkylamines of different chain lengths, hexylamine (C6–CNCs), octylamine (C8–CNCs), and dodecylamine (C12–CNCs). This modification was based on a reductive amination and accompanied by the reduction in the number of sulfate half-ester groups on the surface of the CNCs by ∼50%.^35,36^ The incorporated alkyl groups promoted the formation of a robust self-associated CNC network.^35,36^ The sol–gel transitions of hydrophobized CNCs were reported to happen at lower concentrations than parent sCNCs and the resulted hydrogels with alkylamine groups were extremely strong because of the supramolecular hydrophobic interactions.^35^ Although the number of sulfate half-ester groups was halved, the ζ potential of modified CNCs only decreased by a small amount, and the water contact angle increased from ∼40° to just over 60°.^36^ Accompanied with this modest increase in water contact angle, the surface tension of modified CNC aqueous suspensions decreased as well, indicating the modified CNCs have higher surface activity.^36^ Nevertheless, these CNCs were still dispersible in water.^36^ The binding of hydrophobic groups on the CNC surface decreased the hydrophilicity of the particle’s surface, giving an amphiphilicity to the materials. These modified CNCs, therefore, show promise in stabilizing oil–water interfaces without the need for additional surfactants.

According to Nigmatullin et al.,^36^ CNCs grafted with octylamine (oCNC) have a moderate water contact angle of ∼63° and the surface tension of oCNC aqueous suspensions decreased to ∼51 mN m^–1^. In the present work, oCNCs were produced and used to stabilize linseed oil in water emulsions. These were compared with emulsions stabilized by unmodified sCNCs.

Linseed oil is a natural oil with a high content of glycerol esters of linolenic acid, in which the unsaturated bonds are oxidized when exposed to air. During oxidation, polyunsaturated fatty acids form a three-dimensional network, and the linseed oil gradually dries, exhibiting hardening properties. Because of the drying property, linseed oil has been used in house paints and wood treatments and for the manufacture of various coatings.^37^ Recently, some research has investigated using linseed oil as a healing agent in self-healing composite materials.^12,38−49^ In these works, linseed oil was first made into oil-in-water emulsions and then mixed with resins before polymerization or simply drying.^12,38−48^ Solid particle graphene oxides have been used to stabilize linseed oil in water emulsions by Li et al. before mixing the subsequent microcapsule system with a waterborne polyurethane matrix and drying.^12,38^ In situ polymerization of urea–formaldehyde (UF) resin has been used in a series of works for making stable and durable microcapsules which can be embedded in epoxy resin coatings.^39−48^ These UF–linseed oil microcapsule systems presented both self-healing and anticorrosive properties with healing times varying from 2 days to 30 days and healing temperatures from room temperature to 80 °C.^41−45,48^ A self-healing hydrogel containing CNC (extracted by ethanedioic acid hydrolysis) and linseed oil has also been recently published.^49^

However, no CNMs have been investigated with their efficacy to emulsify linseed oil, nor the fabrication of sustainable self-healing coatings using these materials. Combining linseed oil and CNCs, is a promising approach for making a completely biodegradable and biocompatible self-healing system without the need for chemical synthesis. In this work, we used different concentrations of sCNCs and oCNCs to stabilize linseed oil in water emulsions. After comparing the stability and average oil droplet size of these Pickering emulsions, we selected an emulsion stabilized with 10% oCNCs for manufacturing self-healing coatings. A commercial water-based varnish was readily combined with the emulsion and fabricated into a self-healing coating.

## Experimental Methods

### Materials

Linseed oil (yellow liquid, flash point 113 °C, density 0.93 g/cm^3^ at 25 °C) was bought from Merck Life Science U.K. Ltd. (Dorset, U.K.). 1-Octylamine 99% (molecular formula C_8_H_19_N, boiling point 179 °C) and extra pure ethylene glycol 99+% were purchased from Thermo Fisher Scientific (Lancashire, U.K.). Sodium dodecyl sulfate was purchased from Sigma-Aldrich (Dorset, U.K.). Oxidizing solid potassium periodate 99.8% (230.00 g/mol) was bought from Merck Life Science U.K. Ltd. (Dorset, U.K.). Sodium cyanotrihydridoborate 95% was purchased from Alfa Aesar (Lancashire, U.K.). As it decomposes slowly on exposure to water it was stored carefully in an airtight bottle enclosed with absorbent wool. Freeze-dried CNCs (sodium form) with a 0.94 wt % sulfur content were provided by the Process Development Center, University of Maine (Orono, ME). Dowex Marathon C hydrogen form strong acid cation (SAC) exchange resin was bought from Merck Life Science U.K. Ltd. (Dorset, U.K.). Water-based varnish (gloss) was purchased from the Littlefair’s store (Bristol, U.K.).

### Chemical Modification of CNCs

Freeze-dried sulfated CNCs were modified with octylamine following the procedure by Nigmatullin et al.^35^ In brief, sulfated CNCs were suspended in DI water (1.6 wt %) and reacted with 1.68 mmol of sodium periodate per 1 g of CNCs. After reacting for 48 h, the suspension was dialyzed against DI water overnight. Following dialysis, octylamine was added in the proportion of 7.7 mmol per 1 g of CNCs and left to react at 45 °C for 3 h and another 21 h after addition of sodium cyanotrihydridoborate (40 mM) at room temperature. After this reaction, the product was purified by washing with 2 wt % NaCl in an 2-propanol/water mixture (50/50/v/v) solution and dialyzed against DI water. The concentration of the oCNC aqueous suspension was increased through water evaporation in dialysis tubing until gelation occurred. The final concentration of the oCNC gel was 4.8 wt %.

### Preparation of Pickering Emulsions

Pickering emulsions were prepared by adding 1 g of linseed oil into 15 g of either oCNC or unmodified sCNC suspensions of different concentrations. A range of CNC/oil ratios was targeted; namely 10%, 15%, 20%, 25%, 30%, and 35% of CNCs, with respect to oil. No surfactant was added in the emulsion system and all the emulsions were prepared by sonication using a sonic probe (Branson Digital Sonifer) for 3 min at a 30% amplitude, alternating 5 s of sonication and 5 s rest to prevent boiling. Comparator groups of samples were also produced to better understand the CNCs’ ability to stabilize linseed oil in water. In these samples, a standard surfactant (sodium dodecyl sulfate) or those without an emulsifier were used to stabilize the O/W emulsions. In the third group, samples were made by adding 1 g of linseed oil into 15 g of DI water and mixing using ultrasonication for the same time and power. In the fourth group, sodium dodecyl sulfate was also used to emulsify linseed oil in water. The resultant emulsions were compared with Pickering emulsions stabilized with sCNCs and oCNCs.

### Fourier Transform Infrared (FTIR) Spectroscopy

FTIR spectroscopy was used to distinguish oCNC and sCNC. The same method was used by Nigmatullin et al.^35^ to detect the presence of additional octyl chains on the oCNC surface. A small portion of oCNC gel was dried in a vacuum oven to get the required solid samples. The absorbance of the IR was normalized to a band located at ∼1030 cm^–1^ for both sCNC and oCNC crystalline samples.

### ^1^H–^13^C Cross-Polarization Magic Angle Spinning (CP/MAS) NMR Spectroscopy

Solid-state NMR experiments were performed on a Bruker Avance III NMR spectrometer, equipped with a 4 mm triple resonance probe operating at frequencies of 300.13 MHz (^1^H) and 75.48 MHz (^13^C). oCNC and sulfated CNC powder samples were packed tightly into an 80-μL rotor and spun at a MAS rate of 12 kHz. A ^1^H–^13^C CP/MAS NMR spectrum was acquired at 20 °C using 12k scans, a recycle delay of 10 s, and a contact time of 2 ms. Spectral deconvolution was performed via global spectral deconvolution algorithm using the MestreLab MNova (v14.2) software package.

### Transmission Electron Microscope (TEM)

TEM was used to characterize and measure the lengths and widths of hydrophobized oCNCs. Both FEI Tecnai 12 (120 kV) and FEI Tecnai 20 (200 kV) instruments were used to get high-resolution images. oCNC aqueous suspensions with a concentration of 1 mg/mL were drop-casted onto a carbon-coated electron microscope copper grid and negatively stained with a 2 wt % uranyl acetate solution. These stained samples were dried in an oven overnight before imaging.

### Light Microscopy

Pickering emulsions, using both oCNCs or sCNCs as the stabilizers with a set of CNC/oil ratios, were visualized using optical microscopy. For each sample, 1 mL of the emulsions was diluted with 10 mL of DI water and sonicated using a sonic probe with a 15% amplitude for 1 min to ensure proper mixing. One drop of the diluted emulsion sample was then deposited into a cavity glass slide and visualized with a Zeiss microscope. The sizes of the oil droplets were measured by particle analysis using ImageJ software, and the size distribution was represented graphically by MATLAB software.

### Stability Test

The stability of oil droplets covered by oCNCs was tested during an extended storage period and compared with those stabilized by sCNCs. Photographs of vials were taken over a time period. The thicknesses of the creaming layers were measured using a digital slide calliper. The stability of oCNC emulsions was also tested by centrifugation for 8 min at 6000 rpm. The centrifuged emulsion samples were then visualized by cryo-SEM to obtain the detailed structure of the oil droplets. An optical microscope was used to detect the remaining oil droplets in the separated water phase for both sCNC and oCNC samples.

### Cryo-SEM

High-resolution electron cryo-microscopy was applied to the oCNC Pickering emulsion sample. A Quanta 200 - FEI FEG-SEM, sample preparation equipment, and a data analysis suite were used for these measurements. Emulsions were first rapidly vitrified at a temperature of ∼−140 °C and then fractured to provide fracture surfaces on which both droplet surfaces and their contents were visible. A short sublimation process at ∼−90 °C was required to enhance the presence of the oil droplets at the fracture surface. A thin layer of Au–Pd was coated on the nonconductive fracture surface to prevent charging. The fractured sample was then maintained in liquid nitrogen at ∼−120 °C during the viewing process. The emulsion structure in its native, hydrated state was thereby obtained.

### Self-Healing Coating Preparation

Pickering emulsions with 10% oCNCs were mixed with a water-based varnish (weight ratio 1:1). The mixture was then magnetically stirred at room temperature for 30 min. This mixture was then deposited onto a glass slide and bar coated. Samples were then dried in a vacuum oven at a temperature of 80 °C for 45 min. A vacuum oven was used to prevent the linseed oil from oxidizing during the drying of the coating.

### Self-Healing Test of the Coating

Scratches were made on the surface of the coating with the tip of a metal screw and a scalpel. The coating was then put into an oven at a temperature of 95 °C for 6 h. The air in the oven was kept connected with the outside environment to provide plenty of oxygen. The coating was viewed under both an Olympus microscope and SEM before and after the healing process to detect any physical changes occurring in the region of the scratch.

## Results and Discussion

### OCNC Preparation and Characterization

The oCNCs were modified in aqueous suspension and stored in a bottle after gelation to maintain them in a never-dried state. The gelation process was conducted in dialysis tubing at a moderate temperature (∼45 °C) for more than 48 h. The much lower temperature relative to the boiling point of water protected the modified CNCs from dehydration, and the dialysis tubing, which allowed water vapor to pass through, provided sufficient surface area for water evaporation. No precipitation of the oCNCs was found on the inner wall of the dialysis tubing during the suspension shrinkage process. Conductometric titration tests for both oCNC and sCNC were carried out (see Supporting Information). According to the titration test, the content of -OSO_3_H was 172.2 ± 5.1 mmol kg^–1^ for the oCNC samples and 245.2 ± 10.4 mmol kg^–1^ for sCNC samples (see Supporting Information).

**Figure 1:**
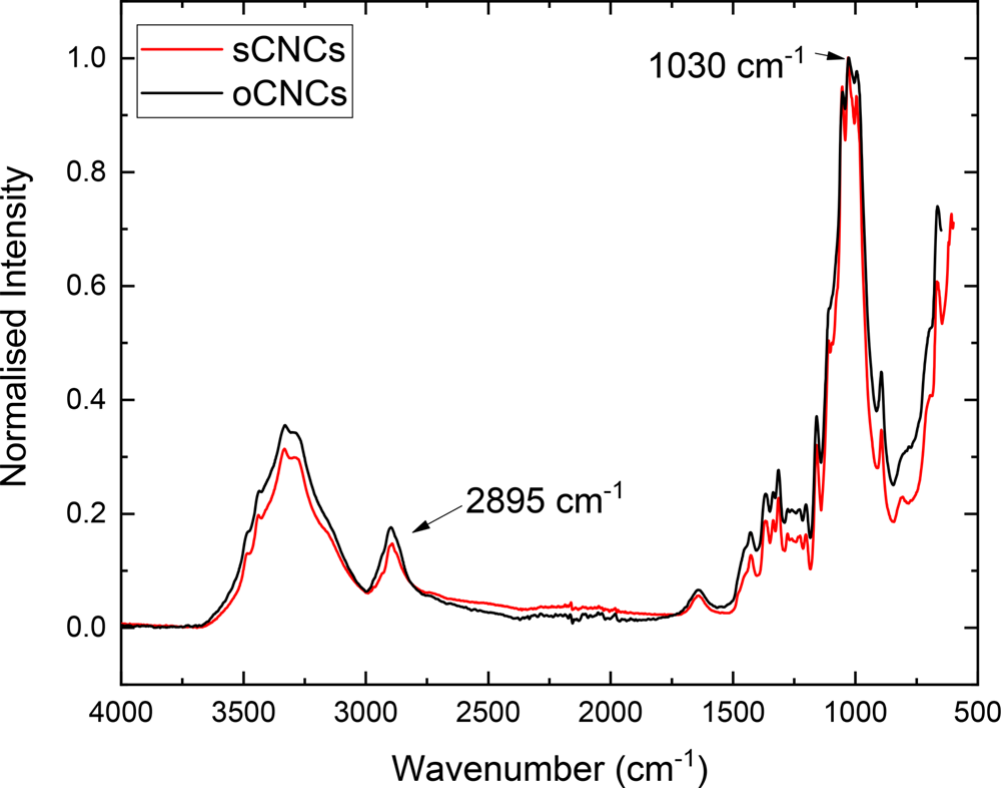
Typical ATR FTIR spectra of sCNC and oCNC (curves shifted to avoid overlap).

The ^1^H–^13^C CP/MAS NMR spectra of oCNC and sCNC demonstrated the presence of octylamine groups on the oCNC surface (Figure S1). The degree of surface functionalization (DSF) of the CNCs was found to be relatively low, in alignment with a previous study.^35^ This was found to be 3%, as detected by nuclear magnetic resonance (NMR); the ratio of the sum of the deconvoluted areas of all octyl peaks (2.5) and the area of the C4 and C6 surface peaks (10.4) was 3% (Figure S2). The presence of grafted octylamine groups was also detected by comparing the normalized absorbance intensity of sCNC and oCNC under Fourier transform infrared spectra (FTIR) as shown in Figure 1. To get clear IR spectra, dehydrated samples were used for both sCNCs and oCNCs. The sCNCs were freeze-dried, as provided by the University of Maine, while the oCNCs were stored in gel form after modification. Oven drying was applied to dehydrate 5 g of the oCNC gel in a vacuum oven at 95 °C for over 2 h and a thin layer oCNC was peeled off after the drying process. The infrared absorbance was normalized at 1030 cm^–1^ for both oCNCs and sCNCs. The maximum intensity of a band at a wavenumber position of 2893 cm^–1^ for sCNCs compared to a position of 2895 cm^–1^ for the oCNCs, which indicated an increase in sp^3^ C–H stretching. This increase in the position and change in intensity (from 0.15 for sCNCs to 0.18 for oCNCs) of this band is thought to be induced by the presence of grafted octylamine groups.

**Figure 2:**
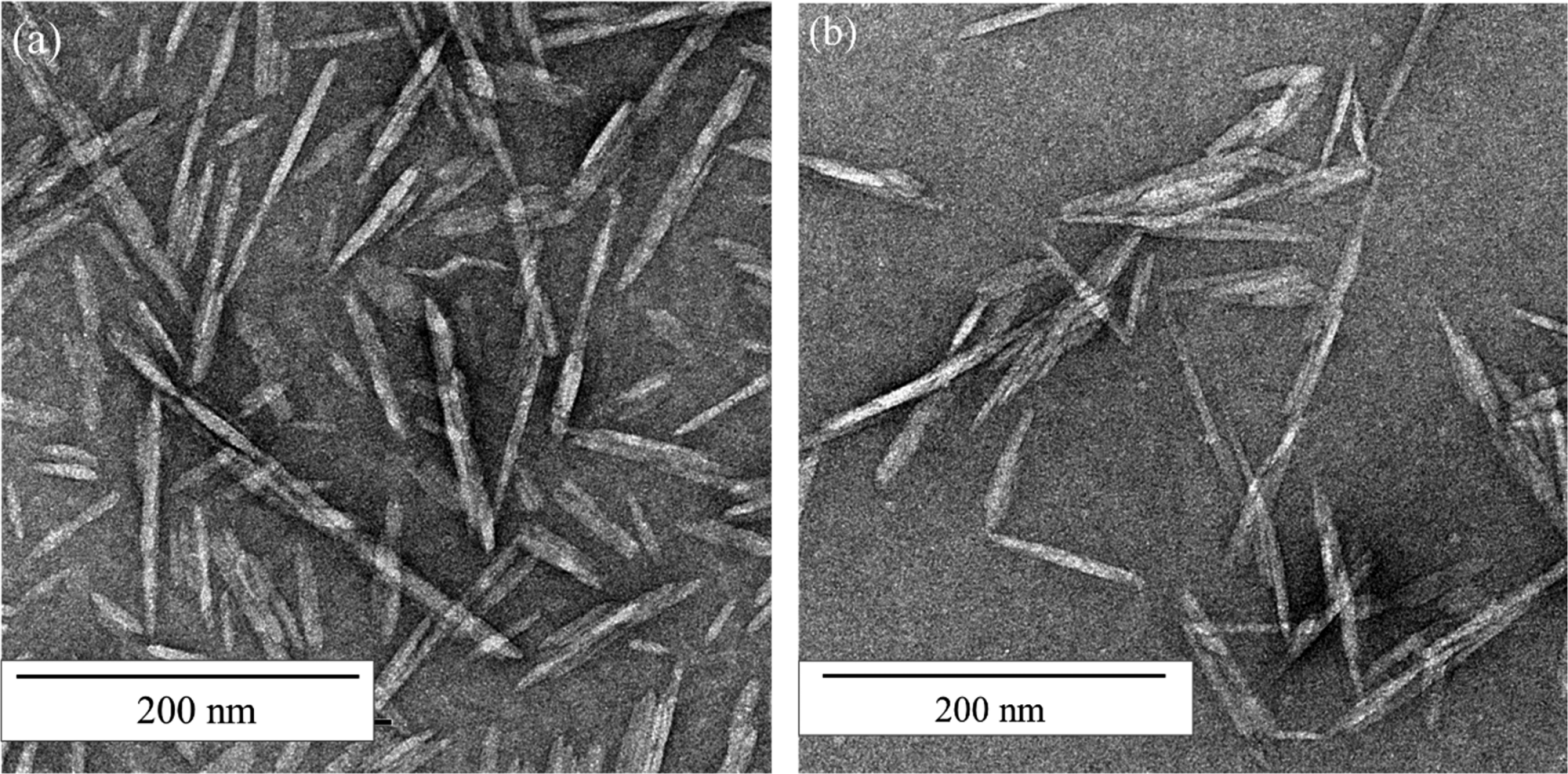
Typical morphology of CNCs imaged using TEM of negative stained CNCs deposited from dilute CNC aqueous suspensions: (a) sCNCs, and (b) oCNCs.

TEM images of CNCs deposited from the 1 mg/mL suspensions (Figure 2) showed that the hydrophobized CNCs have a tendency to aggregate because of van der Waals forces and the hydrophobic interactions between octylamine groups in water.^50^ Analysis of the TEM images showed that the oCNCs have an average length of 168.2 ± 61.6 nm and an average width of 7.2 ± 2.3 nm. The height of the oCNCs was measured to be ∼4 nm in a previous study by atomic force microscopy (AFM).^35^ The modification process did not affect the morphology of the CNCs.

### Emulsion Preparation and Stability Comparison

**Figure 3:**
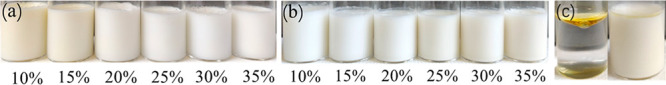
Photograph of Pickering emulsions: (a) sCNC-stabilized emulsions, CNC/oil ratio 10% ∼ 35%; (b) oCNC-stabilized emulsions, CNC/oil ratio 10% ∼ 35%; (c) linseed oil and water mixture without emulsifiers, before and after sonication.

After the 3 min sonication process, linseed oil was dispersed into separate droplets of various sizes, and CNCs were anticipated to adsorb to the oil–water interface. The color of linseed oil under sunlight is yellow, and well-emulsified oil droplets with or without CNCs on the surface are white (Figures 3 and S1 in the Supporting Information). The color difference among the oil-in-water emulsions is a visual representation of the diverse oil droplet sizes.

**Figure 4:**
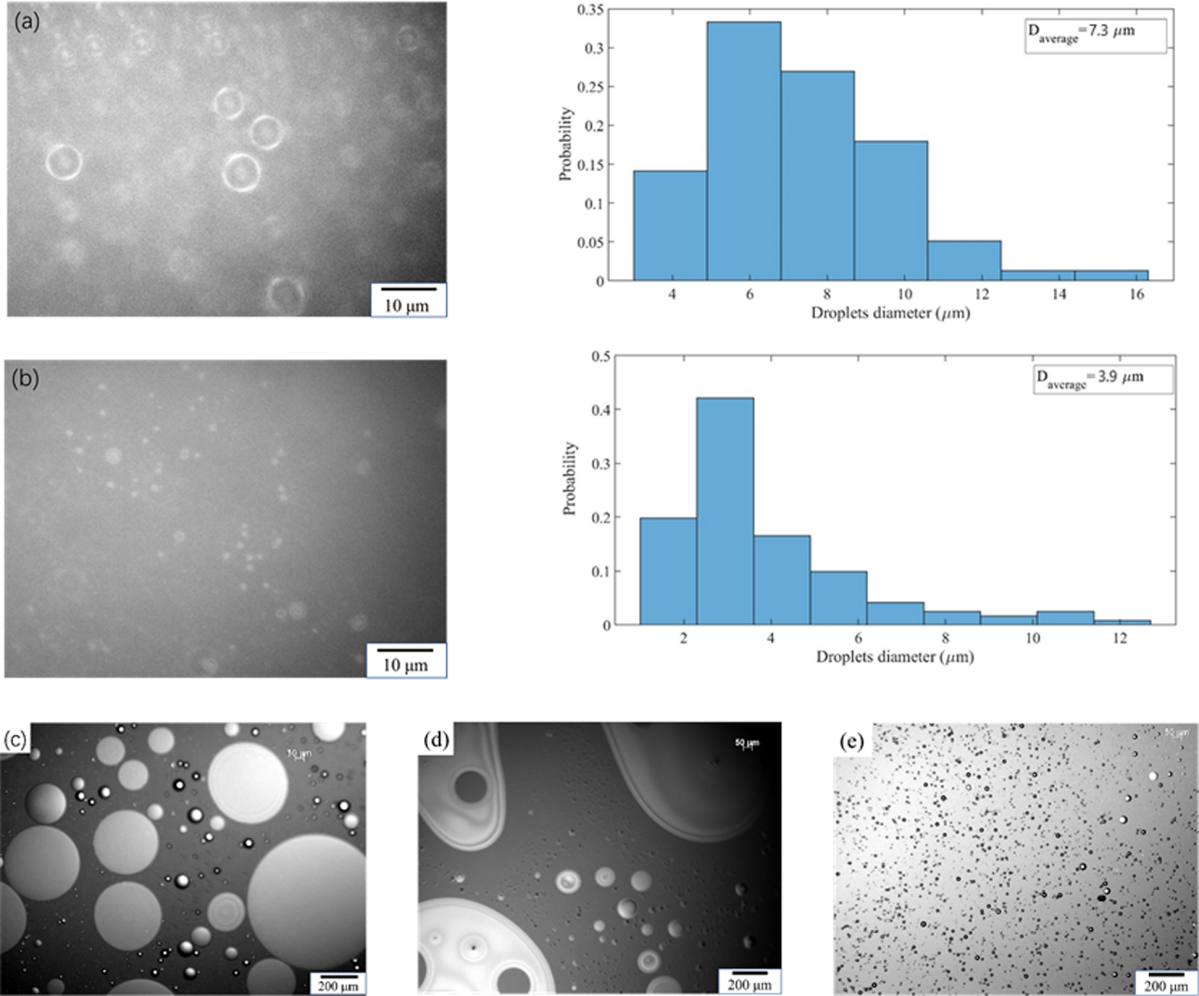
Typical optical microscope images of linseed oil/water emulsions: (a) optical microscope image (left) and droplet diameter histogram of a 20% sCNC-stabilized Pickering emulsion (right), (b) optical microscope image (left) and droplet diameter histogram of a 25% sCNC-stabilized Pickering emulsion (right), (c) linseed oil/water mixture, (d) 10% sCNC-stabilized Pickering emulsion, and (e) optical microscope image of a 10% oCNC-stabilized Pickering emulsion.

For sCNCs, when the CNC/oil ratio is less than 20%, the linseed oil was not completely emulsified after a 3 min ultrasonication. The resulting emulsion was faint yellow in color and an oil residue was found to be floating on the surface (Figure 3a). After increasing the CNC/oil ratio above 20%, the linseed oil was completely emulsified into a milky emulsion and no oil residue was seen on the surface. This change of emulsion color corresponds with the average oil droplet size measurement results obtained from optical microscopy. A large decrease in oil droplet size appeared after the sCNC/oil ratio increased above 20% (Figure 4a,b). As for the oCNCs, after the same ultrasonic processing, the linseed oil was completely emulsified into milky O/W emulsions (Figure 3b) under all studied CNC/oil ratios. No variance in color was seen for the emulsions stabilized with oCNCs when the CNC/oil ratio was altered, and no obvious oil residue was found on the liquid surface. Therefore, oCNCs were more able to stabilize the emulsions than sCNCs at low CNC/oil ratios. In the comparator group, in which no emulsifiers were used, the continuous oil phase was disrupted by strong ultrasonication and a portion of the oil was dispersed in the water phase. The lower water phase was faint yellow (Figure 3c) with a small portion of oil remaining undispersed into the water phase and floating on the surface. Without emulsifiers, the dispersion of oil in water by ultrasonication is nonuniform, and the final mixture does not resemble an emulsion. The oil–water mixtures and the emulsions stabilized with 10% of sCNCs and oCNCs were observed by optical microscopy following sonication (Figure 4c–e). The different dispersions of oil droplets with different emulsifiers (no emulsifier, oCNCs or sCNCs) demonstrated the stabilizing ability of oCNCs and sCNCs. At low concentrations (10 wt % CNC/oil), oCNCs fully stabilized the linseed oil droplets while the sCNCs were insufficient as emulsifiers in this respect. A significant oil residue still existed in the emulsion stabilized with 10% of sCNCs, which presented itself as large irregular shaped droplets (Figure 4d) in contrast to small, dark CNC-stabilized oil droplets. No continuous oil phase was observed for the oCNC emulsion, and small oil droplets were uniformly dispersed in the water phase (Figure 4e).

**Figure 5:**
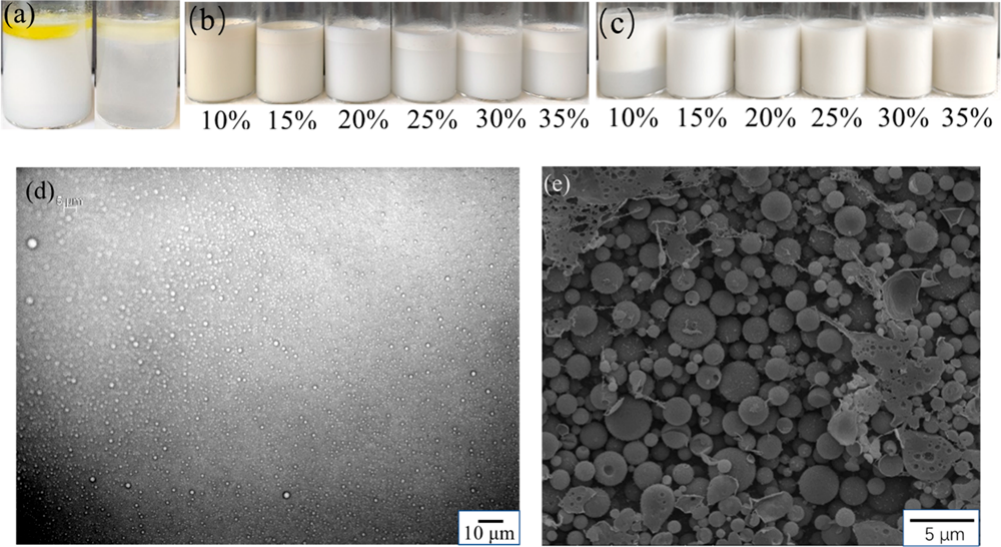
Typical images of the stability test performed on sCNC- and oCNC-stabilized emulsions: photographs of (a) linseed oil and water mixture stored for 24 h (left), and over 72 h (right), (b) 10–35% sCNC-stabilized emulsions stored for 1 h, and (c) 10–35% oCNC-stabilized emulsions stored for 24 h, (d) a typical optical microscope image of a postcentrifugation Pickering emulsion (25% oCNC), and (e) a typical cryo-SEM image of a postcentrifugation Pickering emulsion (25% oCNC).

Emulsion stability can be tested using various methods, including long time storage, centrifugation, and low-intensity ultrasonic vibration or heating.^23^ Emulsions are regarded as stable if the droplets formed can resist physical changes during these tests. To compare the stability between sCNC- and oCNC-stabilized emulsions, long time storage at room temperature and centrifugation at a speed of 6000 rpm was applied. The samples were characterized by taking photographs, by optical microscopy, and by cryo-SEM (Figure 5).

**Figure 6:**
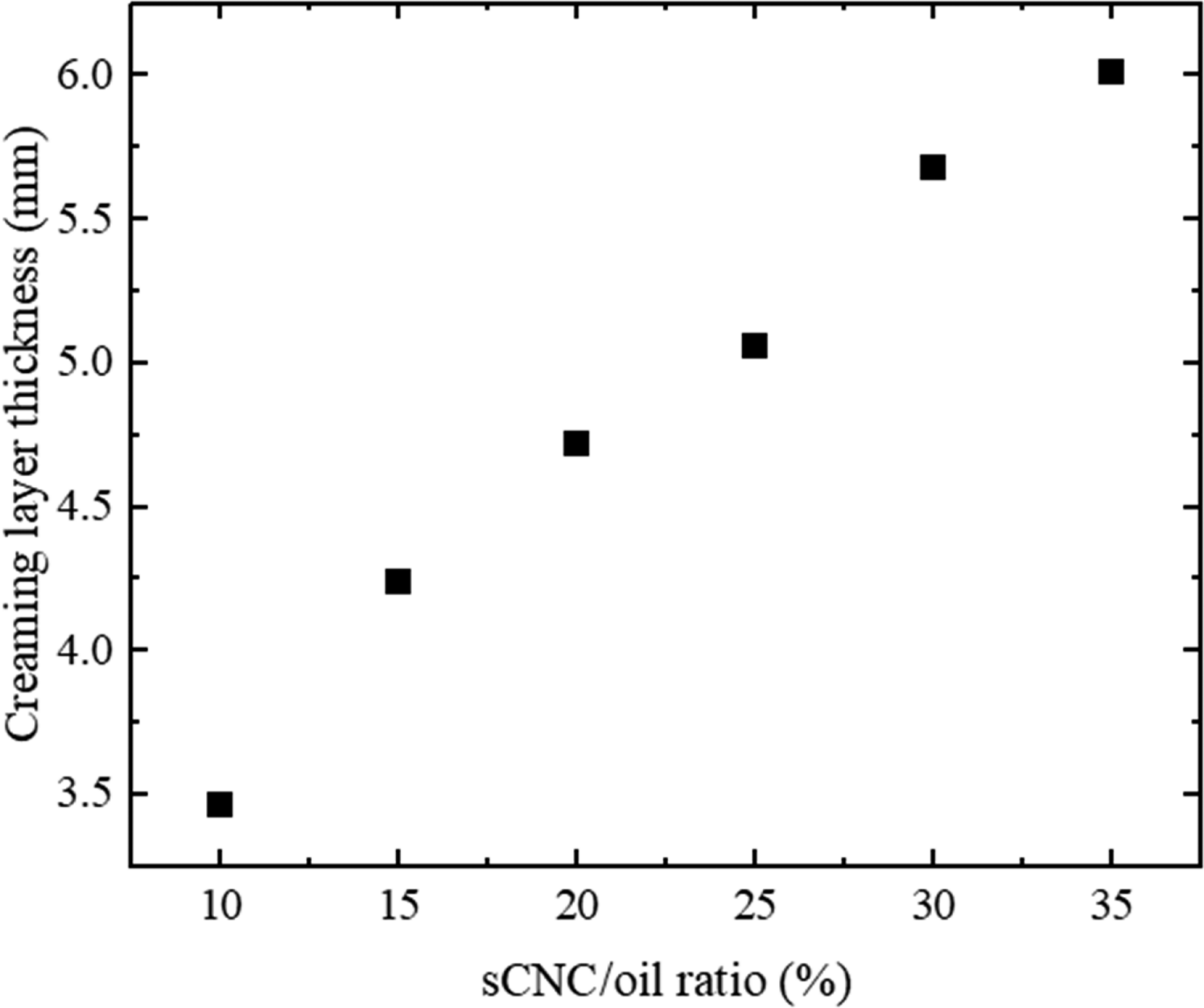
Thickness of the creaming layer for sCNC-stabilized emulsions stored for 1 h at room temperature for different CNC/oil ratios.

The emulsion samples were all stored at room temperature after ultrasonication. Photographs were taken immediately after ultrasonication, 1 h after, and 24 h after, if no obvious changes were observed during the 1-h storage. The oil droplets created by ultrasonication alone were unstable at room temperature. Without the emulsifying effect of CNCs, no creaming of oil droplets was seen in the oil–water mixture, and they coalesced directly into a continuous oil phase and were separated from the continuous water phase. A clear layer of linseed oil was found on the top surface of the mixture after 24 h (Figure 5a). An obvious creaming process was observed for linseed oil droplets in sCNC-stabilized samples, 1 h after sonication (Figure 5b). For all the sCNC samples, with CNC/oil ratios ranging from 10 to 35%, the oil droplets concentrated into a creaming layer and floated to the upper regions of the vials (Figure 5b). The creaming was caused by the low density of CNCs covering linseed oil droplets. The presence of CNCs protected the oil droplets from coalescence. From the optical microscopy and the cryo-SEM images of the postcentrifugation sample (Figure 5d,e), no coalescence of oil droplets was observed for emulsions stabilized with oCNCs. The thickness of the creaming layer increased gradually when the sCNC ratio was increased from 10% to 35% (Figure 6), while the volume of the linseed oil remained unchanged. During room temperature storage, the thickness of the creaming layers did not change while they became increasingly distinguishable from the lower water phase (Figure S4, parts b, d, f, Supporting Information). Similar but faster creaming processes were observed in the emulsion prepared with sodium dodecyl sulfate; the creaming layers were visible on the top of the emulsions within 10 min after ultrasonication (Figure S4g). When the surfactant/oil ratio increased from 20% to 35%, the thicknesses of the creaming layers were always ∼3.5 mm, similar to the Pickering emulsion stabilized with 10% sCNC. Increasing the concentration of sCNCs is thought to be effective in impeding the close aggregation of oil droplets in the creaming layer. The lower water phase, below the creaming layer, remained opaque, and a few oil droplets were detected by optical microscopy (Figure S5, Supporting Information). A fast creaming process has also been reported for bacterial CNC-stabilized hexadecane/water emulsions,^23^ however, the emulsions’ resistance to creaming was not discussed.

All the emulsions formed using oCNCs retained stable oil droplets which were uniformly dispersed in the water phase over a relatively short period of time (1 h). This dispersion of oil droplets remained unchanged for another 24 h in samples with oCNC/oil ratios greater than 10%, while a creaming layer was formed in the emulsion with the lowest oCNCs content (10%) (Figures 5c and S4e, Supporting Information). The creaming layer of oCNC-stabilized emulsions was ∼16 mm, which was much thicker than those stabilized with sCNCs (∼4 mm for a 10% sCNC-stabilized emulsion), while the excess water phase below the creaming layer was only ∼9 mm high. The thickness or volume of the creaming layer illustrated the extent of the compaction of the oil droplets; a thin creaming layer indicates a close packing of the droplets and vice versa. Enhanced resistance to creaming both in time and physical scales was apparent for the oCNC-stabilized emulsions. The presence of octylamine groups is thought to delay the creaming process by increasing the viscosity of the continuous phase^3^ and by resisting the aggregation of oil droplets through osmotic repulsive forces between CNCs.^50^ As measured in previous studies,^35,36^ oCNCs have a degree of surface functionalization (DSF) of only 4 ± 0.1%, and the ζ potential remained almost unchanged compared with sCNCs, so that the enhanced resistance to creaming is not thought to be caused by electrostatic interactions among CNCs. In contrast, the steady flow viscosity of unmodified CNC aqueous suspensions has been shown to be much lower than hydrophobized oCNC systems.^36^ The amount of CNCs added in the emulsification process exceeded the amount actually covering the oil–water interfaces, and it is thought that this excess, dispersed in the continuous water phase, helped to retard the creaming process. The dramatic reduction in the creaming rate and its extent shown in oCNC-stabilized emulsions is thought to be mostly on account of the enhanced viscosity of the oCNC aqueous suspensions, as has previously been suggested.^3,4^

Centrifugation was conducted on the oCNC-stabilized emulsions to further examine their ability to resist creaming at different CNC/oil ratios. The speed of centrifugation was 6000 rpm and the time set to 8 min. For all the emulsions, a small portion of oil droplets was found to be attached to the inner wall of the centrifuge tubes because they were mounted inclined to the center of the instrument. During the centrifugation, the oil droplets were pressed to the wall by water which is larger in mass and inertia. After centrifugation, the tubes were inverted when creaming, and separation between excess water and creaming layers occurred in the emulsions with CNC/oil ratios less than 25%. Compared with the long-time-storage group, the excess water phase in the centrifuge tubes was more transparent and the thicknesses of the creaming layers were thinner (Figure S6, Supporting Information). It was revealed that the aggregation of oil droplets under high-speed centrifugation was more intense. However, for the emulsions with oCNC/oil ratios of more than 25%, centrifugation at 6000 rpm was still not strong enough to outweigh the stabilizing effect provided by the octylamine groups on the CNC surfaces. In these samples, there was no creaming layer, and a water phase separation was not observed.

**Figure 7:**
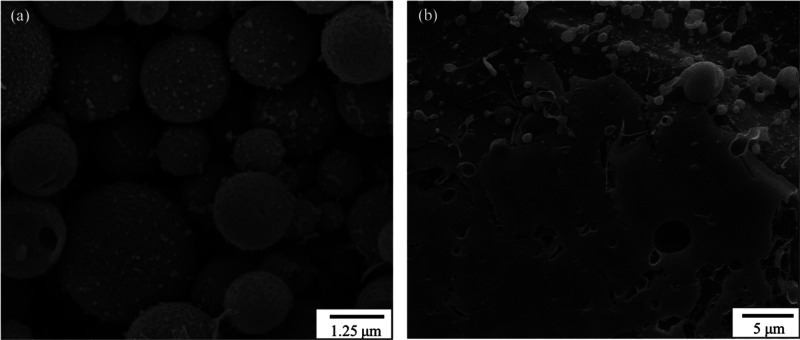
Typical cryo-SEM images of the O/W Pickering emulsions stabilized by oCNCs: (a) vitrified oil droplets and (b) broken droplets at the fracture surface and cavities remaining.

Cryo-SEM was used to characterize the detailed physical changes in the morphology of oil droplets during centrifugation (Figure 7). In the postcentrifugation sample, oil droplets were found to have compacted closely with each other. At some of the interfaces between contacting droplets, there was no notable coalescence, and the oil droplets maintained their spherical shape (Figure 7a). White floccules seen in the SEM images are most likely to be frost induced in the temperature decrease–raise–decrease process, as has been previously reported.^51^ Faint images of oCNC network structures were observed on the outer surfaces of the oil droplets (Figure 7a). It is thought that individual CNCs are not visible in the hydrated state of the Pickering emulsions because of the poor contrast between CNCs and the background linseed oil and water, the small dimensions of oCNCs/sCNCs and the resolution limit of the microscope. Clear nanocrystal network structures have been observed on the surfaces of polymerized latex particles produced from BCN- or CNC-stabilized Pickering emulsions.^23,27^ In these previous studies, the average length of the BCNs presented on polymerized latex particles was 855 nm^23^ which is much longer than the oCNCs/sCNCs used in this work and therefore easier to observe. The average length of the CNCs used by Zhang et al. was about 300 nm and the resolution of the SEM they used was much higher.^27^ In both studies, the network structure of CNMs was observed with dried and solidified latex beads as the background. It is also worth pointing out that cryo-SEM is different to standard electron microscopy, and previous work has shown that individual cellulose chains at the droplet surface are also not visible in nonpolymerized particles.^52^

At the fracture surface of the vitrified emulsions, both unbroken and broken oil droplets were observed. For broken oil droplets, the linseed oil had sublimated, and empty cavities therefore remained (Figure 7b).

### Droplet Characterization

**Figure 8:**
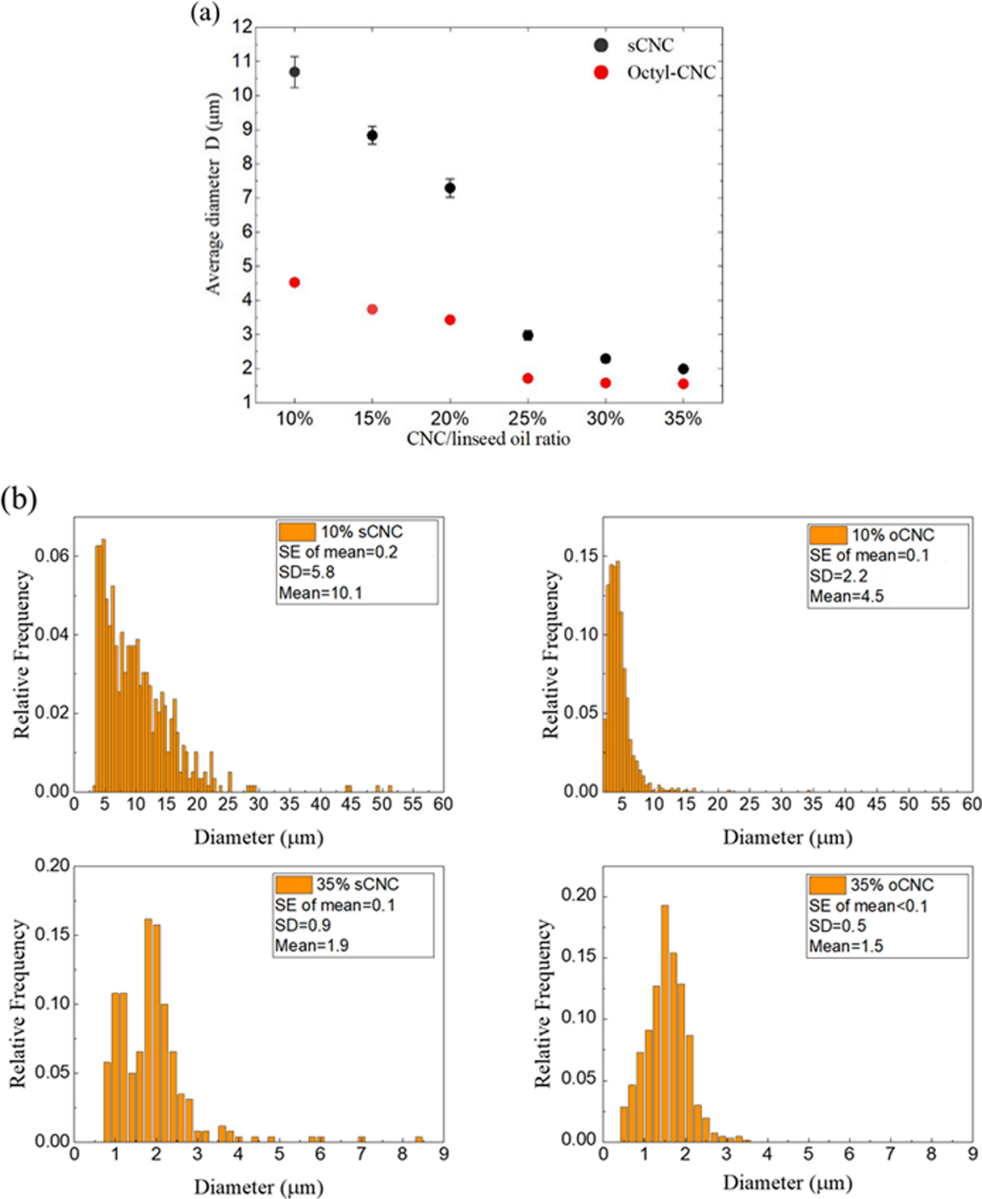
(a) Average size of linseed oil droplets as a function of the CNC/linseed oil ratio in Pickering emulsions. (b) Droplet size distributions of 10% sCNC-, 10% oCNC-, 35% sCNC-, and 35% oCNC-stabilized emulsions. Data shown for both sulfated (sCNC) and octylamine (oCNC) modified cellulose nanocrystals (CNC). Error bars are standard errors from the mean (SE), derived from the standard deviation (SD).

The size of oil droplets was measured from optical microscope images (see Figure 4). The emulsions stabilized with either sCNC or oCNC were diluted with DI water before viewing, after which the distances between droplets were increased. Interval graphs of the average oil droplet diameters (Figure 8) and its reciprocal versus the CNC/oil ratio were plotted (Figure S7, Supporting Information). For each data point, more than 200 oil droplets’ sizes were measured.

For the linseed oil droplets stabilized by both sCNCs or oCNCs, the average diameter decreases with an increase in the CNC/oil ratio. For oCNC-stabilized oil droplets, the diameter decreased to a plateau of ∼2 μm for all the samples with CNC/oil ratios higher than 25%. A similar plateau of average diameter at 2.0 ± 0.1 μm was found for sCNC-stabilized emulsions with CNC/oil ratios higher than 30%. A decrease in the average droplet area is thought to be due to the lowest free energy principle, which has been previously interpreted by the “limited coalescence process”.^4,23^ During the sonication process, a much larger oil–water interface than the CNCs could possibly cover is produced, and the area of this interface decreases in the form of a coalescence of droplets to reduce the total free energy when the sonication is ended. Since the CNCs are adsorbed to the interface they reduce the strong interfacial tension between the oil and the water phase.^3^

The shrinking of this interface and the coalescence of droplets ends when all the remaining interfaces are sufficiently covered with CNCs. Increasing the CNC concentration eventually provides more interfacial area that can be covered, and therefore, fewer droplets coalesced after sonication.

The lower limit of the droplet size is thought to be predicated by the size and flexibility of solid particles,^23^ in this case CNCs. Without sufficient CNC particles in the continuous phase, these small oil droplets rapidly coalesce into larger droplets, and once the CNC concentration reaches a critical point, enough CNC particles are adsorbed to the small oil droplets’ interfaces, thereby inducing an decrease in the average droplet size. For both sCNCs and oCNCs, the sudden drop in droplet size happened at a CNC/oil ratio of 25%, and the lower limits of droplet size stabilized for both circumstances were close, and consistent with the theoretically similar shape and size of two kinds of CNCs. However, the difference in CNC surface groups may also influence the lower limit of droplet size and may cause a small gap of approximately 0.5 μm between the plateau values.

At all the studied CNC concentrations, the average size of oCNC-stabilized oil droplets was smaller than the sCNC-stabilized ones. The difference between the average droplet size stabilized with sCNCs and oCNCs decreased with an increase in the CNC/oil ratio. The difference in droplet sizes is in accordance with the conclusions from previous studies^3,7^ that particles with intermediate wettability can provide stabler emulsions with smaller oil droplets. The octylamine-modified CNCs have been found to be more hydrophobic than sCNCs,^36^ and accordingly they therefore produce smaller O/W emulsion droplets.

The error bars (standard errors from the mean) in Figure 8a are so small that they cannot be seen in the figure, especially for the oCNC-stabilized samples and the samples with a high concentration of sCNCs. A decreasing tendency of the standard errors from the means (SEs) is presented for sCNC samples as the sCNC concentration increases. Four oil droplet diameter histograms for the samples with 10% and 35% sCNC (or oCNC)/oil ratio are presented in Figure 8b, which demonstrated the SEs, standard deviations (SDs), and mean values at each condition. Both the SEs and SDs decrease with the increase in the CNC:oil ratio for either sCNC or oCNC. oCNCs are also able to provide a narrower oil droplet size distribution (smaller SEs and SDs for each CNC/oil ratio) compared with sCNCs, which corresponds with the size of the error bars in Figure 8a.

A simple estimation of the theoretical coverage of CNCs on the droplet surface can be expressed using the equation
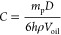
1where *m*_*p*_ is the mass of CNCs adsorbed to the interface, *D* is the average oil droplet size, *h* is the CNC thickness, ρ is the CNC bulk density, and *V*_oil_ is the volume of oil included in the emulsion.^23,27^ The estimation was made based on several assumptions, including that all the CNCs added to the aqueous suspension were adsorbed to the water–oil interface, and the volume of the oil included in the emulsion is equal to the volume of the creaming layer after centrifugation or to the total volume of oil added at the beginning of the experiment. However, these assumptions were not satisfied in our sCNC- and oCNC-stabilized samples. As shown in parts b and c of Figure 5, the volume of the creaming layer was affected by the concentration and surface functional groups of CNCs, and not all the linseed oil was emulsified in the low-concentration sCNC samples. The volume of oil included in the emulsions with an sCNC/oil ratio of 10% or 15% was hard to estimate. More importantly, our investigated CNC/oil ratio ranges from 10% to 35% by weight, much higher than the CNC/oil ratios previously investigated,^23,27^ where the CNC coverage was estimated by eq 1. Their calculated coverage exceded 100% when the CNC concentration was higher than 9 mg per 1 mL of oil, while the CNC concentration in our study is always higher than 93 mg per 1 mL of oil (calculated from the lowest CNC/oil weight ratio, 10%, and the density of linseed oil, 0.93 g/mL). Excess CNCs were dispersed in the continuous phase resisting the creaming of oil droplets; therefore, the adsorbed CNC mass was hard to calculate. However, with CNC concentrations higher than 93 mg per 1 mL of oil, we can estimate that the coverage of CNC at all the emulsified oil droplet interfaces remained unchanged at a theoretically high value. Equation 1 can be transformed to
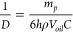
2where the thickness of CNCs *h* and the density of CNC ρ are constant. Assuming the volume of oil included in the emulsion *V*_*oil*_ and CNC coverage *C* remain unchanged in all the samples, eq 2 can be simplified to the relationship
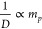
3According to the reciprocal of average diameter of oil droplets, we compare the mass of CNCs adsorbed to the oil–water interface in all the samples, as shown in Figure S4.

With an increase in the CNC concentration, more CNCs are adsorbed to the oil–water interfaces during the sonification process, which was predicted by the “limited coalescence process”. The increasing rate of adsorbed CNCs remains almost constant in the CNC/oil ratio range ∼10%–20%, and suddenly there is an decrease in the droplet size at a critical point of ∼25% for both sCNCs and oCNCs. This sudden change is consistent with the sharply decreased droplet size shown in optical microscope images (Figure 4a,b). For both CNCs, the adsorbed CNC mass should reach a plateau that is limited by the flexibility and length of the rod-like particles.^23^ Similar critical points for both CNCs are indirect confirmation that chemical modification did not affect aspect ratio of CNCs. A clear plateau appeared for CNC/oil ratios above 30% for both CNC samples.

## Characterization of a Self-Healing Coating

**Figure 9:**
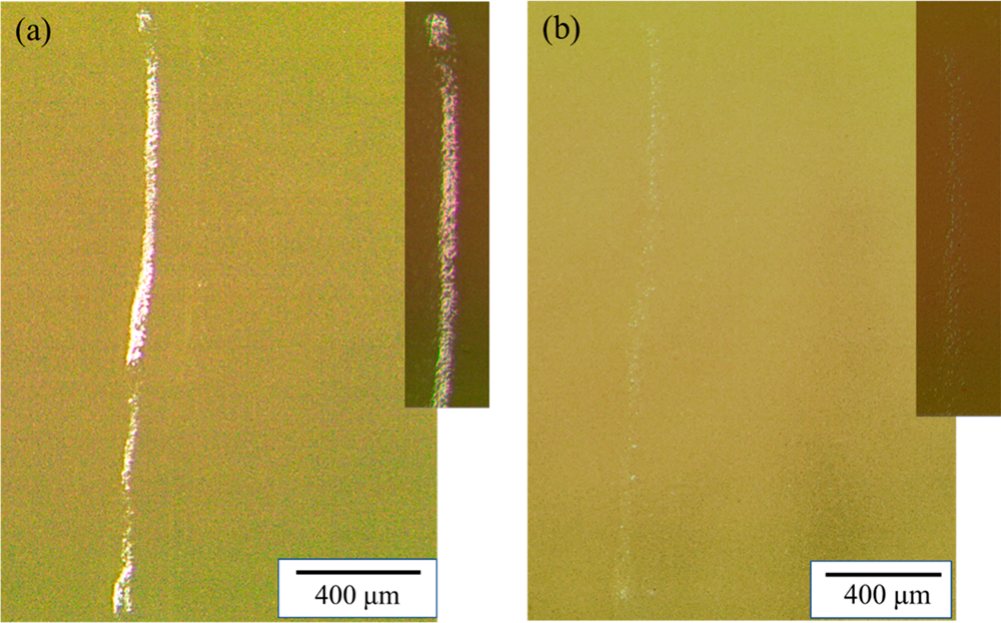
Typical optical microscope images of a scratch on the coating (a) before healing and (b) after healing.

The water-based varnish was mixed with a 10% oCNC-stabilized Pickering emulsion. These varnishes are typically used to protect materials like wood from degradation, and so a self-healing coating could add further functionality to this. The emulsion can resist creaming for over 1 h (Figure 5), and the oil droplets inside are the largest among all the oCNC-stabilized samples (Figure 8), which should readily fracture when a scratch is formed. The mixture formed a uniform coating on the glass slides, where all the oil droplets were well dispersed in the coating and no aggregation of oil droplets was observed under the optical microscope (Figure 9).

From the optical microscope images, the principle of the self-healing system is demonstrated. When the coating was scratched, the oil droplets fracture allowing linseed oil to leak into the gap (Figure 9a). The leaked linseed oil refracts the light which presents itself in a color change in the optical microscope. This color change alone indicates the fracture of oil droplets and exposure of liquid oil at the scratch surface. After exposure to the air for 6 h at a temperature of 95 °C, the linseed oil oxidized and dried within the scratch, filling the gap and healing the scratch (Figure 9b). Because the material at the healed scratch (oxidized linseed oil) is different from that of the surrounding areas (polyurethane varnish), the healed scratch is still visible under the optical microscope because of the different optical performance of the materials. SEM was also used to detect the self-healing of the coating. A scalpel was used instead of a metal screw to cut the coating as it can provide a regular and uniform scratch, suitable for imaging in the SEM. A conductive coating is needed for the imaging in the SEM, which may interfere with the leakage and oxidization of the linseed oil. Therefore, the self-healing sample was only viewed under the SEM after healing and another control group without any linseed oil was also scratched and used as a control. Scratches were made with the same scalpel for both groups, and the control group (pure polyurethane varnish on glass slide) was also put into the oven together with the self-healed samples for 6 h.

**Figure 10:**
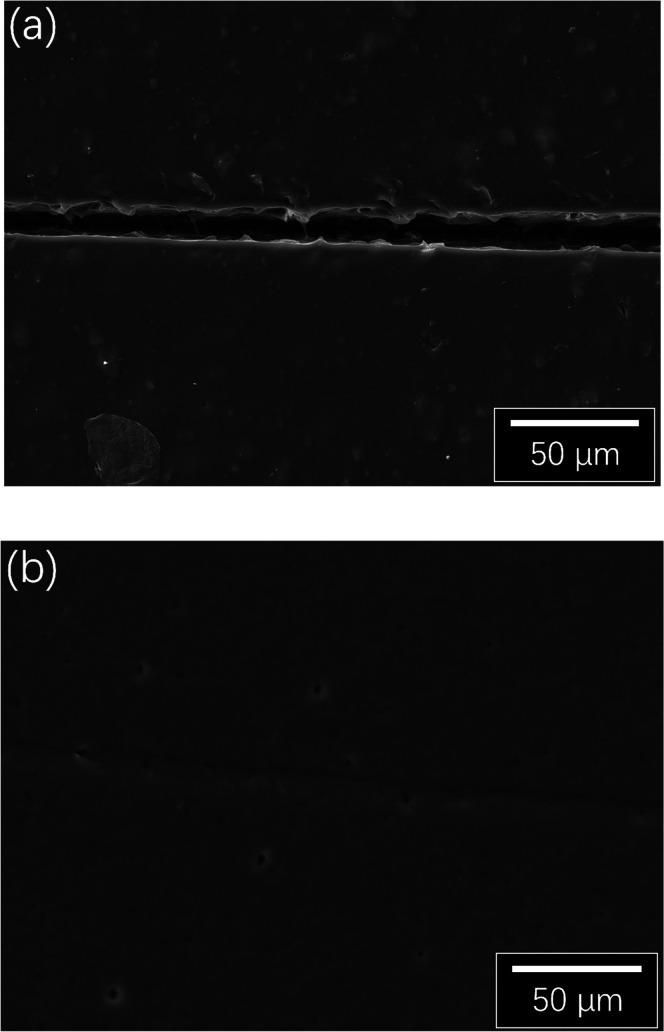
Typical SEM images of (a) a scratch on the pure varnish coating and (b) a scratch on the self-healed coating after heating. The brightness of the images has been adjusted for clarity.

For the pure varnish coating, the width of the scalpel-made scratch is ∼12 μm (Figure 10a), and both the edges and gap of the scratch are clearly visible. In comparison, the scratch can hardly be seen on the coating with linseed–CNC droplets (Figure 10b) after healing and the existence of microscale oil droplets are also visible. The healing effect presented by SEM images concur with Wang and Zhou’s study, in which an epoxy coating with poly(urea–formaldehyde) (PUF) capsulated linseed oil was healed at room temperature for 5 days followed by heating in an oven for 4 h at 80 °C.^48^ Our study has shown that healing can occur at similar temperatures, but with much shorter curing times.

## Conclusions

Both sCNCs and oCNCs have been demonstrated to be able to stabilize linseed oil in a continuous water phase. The grafted octylamine groups on the oCNC surfaces improved the oil droplets’ resistance to creaming and decreased the average size of the oil droplets. By increasing the CNC concentration in the continuous water phase, the oil droplets stabilized by both sCNCs and oCNCs became smaller. The decrease in size reached a plateau when the CNC/oil ratio exceeded 30%. A CNC network around the oil droplets acted as a protector that prevented the oil droplets from coalescing or collapsing during long-time storage and high-speed centrifugation. It was demonstrated that such Pickering emulsions can be further mixed with water-based formulations without inducing oil droplet coalescence. Emulsions stabilized with 10% oCNC were combined with a commercial water-based varnish resulting in coatings, which exhibited an ability to heal various types of scratches at 95 °in a short period of time. Thus, the approach based on Pickering emulsions stabilized with hydrophobized CNCs provides an autocatalytic self-healing coating that could be applied to a range of materials that need oxidative protection, e.g., wood, metals, etc. Given its applicability to a low-density oil like linseed, there is also potential to extend this work to other oils.

## Supporting Information Available

Figure S1, ^1^H–^13^C CP/MAS NMR spectral of CNCs; Figure S2, NMR spectral deconvolution of oCNC; Figure S3, conductivity titration results; Figure S4, photographs of emulsions; Figure S5, optical microscope of oil droplet residues in a separated water phase; Figure S6, photographs of oCNC emulsions after centrifugation; Figure S7, reciprocal of droplet average diameter vs CNC/oil ratio (PDF)

## Abbreviations

O/W: oil in water
CNM: cellulose nanomaterial
MC: methyl cellulose
HPMC: hydroxypropyl methyl cellulose
BCN: bacterial nanocrystal
CNC: cellulose nanocrystal
AcCNF: acetylated cellulose nanofibril
MMA: methyl methacrylate
sCNC: sulfated cellulose nanocrystal
oCNC: octylamine cellulose nanocrystal
DBSA: dodecylbenzenesulfonic acid
DI water: deionized water
FTIR: Fourier transform infrared
TEM: transmission electron microscope
SEM: scanning electron microscope
DSF: degree of surface functionalization
NMR: nuclear magnetic resonance
AFM: atomic force microscopy

## References

[ref1] Pickering S. U. CXCVI.—Emulsions. J. Chem. Soc., Trans. 1907, 91 (0), 2001–2021. 10.1039/CT9079102001.

[ref2] Aveyard R.; Binks B. P.; Clint J. H. Emulsions Stabilised Solely by Colloidal Particles. Adv. Colloid Interface Sci. 2003, 100–102, 503–546. 10.1016/S0001-8686(02)00069-6.

[ref3] Binks B. P. Particles as Surfactants - Similarities and Differences. Curr. Opin. Colloid Interface Sci. 2002, 7 (1–2), 21–41. 10.1016/S1359-0294(02)00008-0.

[ref4] Binks B. P.; Lumsdon S. O. Pickering Emulsions Stabilized by Monodisperse Latex Particles: Effects of Particle Size. Langmuir 2001, 17 (15), 4540–4547. 10.1021/la0103822.

[ref5] Zhang Y.; Yang H.; Naren N.; Rowan S. J. Surfactant-Free Latex Nanocomposites Stabilized and Reinforced by Hydrophobically Functionalized Cellulose Nanocrystals. ACS Applied Polymer Materials 2020, 2 (6), 2291–2302. 10.1021/acsapm.0c00263.

[ref6] Amalvy J. I.; Armes S. P.; Binks B. P.; Rodrigues J. A.; Unali G. F. Use of Sterically-Stabilised Polystyrene Latex Particles as a pH-Responsive Particulate Emulsifier to Prepare Surfactant-Free Pil-in-Water Emulsions. Chem. Commun. 2003, (15), 1826–1827. 10.1039/b304967a. 12931986

[ref7] Binks B. P.; Whitby C. P. Nanoparticle Silica-Stabilised Oil-in-Water Emulsions: Improving Emulsion Stability. Colloids Surf., A 2005, 253 (1), 105–115. 10.1016/j.colsurfa.2004.10.116.

[ref8] Binks B. P.; Lumsdon S. O. Stability of Oil-in-Water Emulsions Stabilised by Silica Particles. Phys. Chem. Chem. Phys. 1999, 1 (12), 3007–3016. 10.1039/a902209k.

[ref9] Abend S.; Lagaly G. Bentonite and Double Hydroxides as Emulsifying Agents. Clay Minerals 2001, 36 (4), 557–570. 10.1180/0009855013640009.

[ref10] Ashby N. P.; Binks B. P. Pickering Emulsions Stabilised by Laponite Clay Particles. Phys. Chem. Chem. Phys. 2000, 2 (24), 5640–5646. 10.1039/b007098j.

[ref11] Bhagavathi Kandy S.; Simon G. P.; Cheng W.; Zank J.; Joshi K.; Gala D.; Bhattacharyya A. R. Effect of Incorporation of Multiwalled Carbon Nanotubes on the Microstructure and Flow Behavior of Highly Concentrated Emulsions. ACS Omega 2018, 3 (10), 13584–13597. 10.1021/acsomega.8b00579. 31458064 PMC6644587

[ref12] Li J.; Li Z.; Feng Q.; Qiu H.; Yang G.; Zheng S.; Yang J. Encapsulation of Linseed Oil in Graphene Oxide Shells for Preparation of Self-Healing Composite Coatings. Prog. Org. Coat. 2019, 129, 285–291. 10.1016/j.porgcoat.2019.01.024.

[ref13] Kim S. D.; Zhang W. L.; Choi H. J. Pickering Emulsion-Fabricated Polystyrene–Graphene Oxide Microspheres and Their Electrorheology. J. Mater. Chem. C 2014, 2 (36), 7541 10.1039/C4TC01040J.

[ref14] Melle S.; Lask M.; Fuller G. G. Pickering Emulsions with Controllable Stability. Langmuir 2005, 21 (6), 2158–2162. 10.1021/la047691n. 15752002

[ref15] Qiao X.; Zhou J.; Binks B. P.; Gong X.; Sun K. Magnetorheological Behavior of Pickering Emulsions Stabilized by Surface-Modified Fe3O4 Nanoparticles. Colloids Surf., A 2012, 412, 20–28. 10.1016/j.colsurfa.2012.06.026.

[ref16] Laredj-Bourezg F.; Chevalier Y.; Boyron O.; Bolzinger M.-A. Emulsions Stabilized with Organic Solid Particles. Colloids Surf., A 2012, 413, 252–259. 10.1016/j.colsurfa.2011.12.064.

[ref17] Binks B. P.; Clint J. H.; Mackenzie G.; Simcock C.; Whitby C. P. Naturally Occurring Spore Particles at Planar Fluid Interfaces and in Emulsions. Langmuir 2005, 21 (18), 8161–8167. 10.1021/la0513858. 16114917

[ref18] Fujii S.; Aichi A.; Muraoka M.; Kishimoto N.; Iwahori K.; Nakamura Y.; Yamashita I. Ferritin as a Bionano-Particulate Emulsifier. J. Colloid Interface Sci. 2009, 338 (1), 222–228. 10.1016/j.jcis.2009.06.028. 19604513

[ref19] van Rijn P.; Mougin N. C.; Franke D.; Park H.; Böker A. Pickering Emulsion Templated Soft Capsules by Self-Assembling Cross-Linkable Ferritin–Polymer Conjugates. Chem. Commun. 2011, 47 (29), 8376–8378. 10.1039/c1cc12005k. 21687903

[ref20] Han S.; Lyu S.; Chen Z.; Fu F.; Wang S. Combined Stabilizers Prepared From Cellulose Nanocrystals and Styrene-Maleic Anhydride to Microencapsulate Phase Change Materials. Carbohydr. Polym. 2020, 234, 115923 10.1016/j.carbpol.2020.115923. 32070542

[ref21] Shi X.; Yazdani M. R.; Ajdary R.; Rojas O. J. Leakage-Proof Microencapsulation of Phase Change Materials by Emulsification With Acetylated Cellulose Nanofibrils. Carbohydr. Polym. 2021, 254, 117279 10.1016/j.carbpol.2020.117279. 33357855

[ref22] Kedzior S. A.; Dubé M. A.; Cranston E. D. Cellulose Nanocrystals and Methyl Cellulose as Costabilizers for Nanocomposite Latexes with Double Morphology. ACS Sustainable Chem. Eng. 2017, 5 (11), 10509–10517. 10.1021/acssuschemeng.7b02510.

[ref23] Kalashnikova I.; Bizot H.; Cathala B.; Capron I. New Pickering Emulsions Stabilized by Bacterial Cellulose Nanocrystals. Langmuir 2011, 27 (12), 7471–7479. 10.1021/la200971f. 21604688

[ref24] Kolanowski W.; Laufenberg G.; Kunz B. Fish Oil Stabilisation by Microencapsulation With Modified Cellulose. International Journal of Food Sciences and Nutrition 2004, 55 (4), 333–343. 10.1080/09637480410001725157. 15369987

[ref25] Drelich A.; Gomez F.; Clausse D.; Pezron I. Evolution of Water-in-Oil Emulsions Stabilized With Solid Particles: Influence of Added Emulsifier. Colloids Surf., A 2010, 365 (1), 171–177. 10.1016/j.colsurfa.2010.01.042.

[ref26] Binks B. P.; Rodrigues J. A.; Frith W. J. Synergistic Interaction in Emulsions Stabilized by a Mixture of Silica Nanoparticles and Cationic Surfactant. Langmuir 2007, 23 (7), 3626–3636. 10.1021/la0634600. 17316038

[ref27] Zhang Y.; Karimkhani V.; Makowski B. T.; Samaranayake G.; Rowan S. J. Nanoemulsions and Nanolatexes Stabilized by Hydrophobically Functionalized Cellulose Nanocrystals. Macromolecules 2017, 50, 6032–6042. 10.1021/acs.macromol.7b00982.

[ref28] Foster E. J.; Moon R. J.; Agarwal U. P.; Bortner M. J.; Bras J.; Camarero-Espinosa S.; Chan K. J.; Clift M. J. D.; Cranston E. D.; Eichhorn S. J.; Fox D. M.; Hamad W. Y.; Heux L.; Jean B.; Korey M.; Nieh W.; Ong K. J.; Reid M. S.; Renneckar S.; Roberts R.; Shatkin J. A.; Simonsen J.; Stinson-Bagby K.; Wanasekara N.; Youngblood J. Current Characterization Methods for Cellulose Nanomaterials. Chem. Soc. Rev. 2018, 47 (8), 2609–2679. 10.1039/C6CS00895J. 29658545

[ref29] Delepierre G.; Eyley S.; Thielemans W.; Weder C.; Cranston E. D.; Zoppe J. O. Patience is a Virtue: Self-Assembly and Physico-Chemical Properties of Cellulose Nanocrystal Allomorphs. Nanoscale 2020, 12 (33), 17480–17493. 10.1039/D0NR04491A. 32808640

[ref30] Fujisawa S.; Togawa E.; Kuroda K. Facile Route to Transparent, Strong, and Thermally Stable Nanocellulose/Polymer Nanocomposites from an Aqueous Pickering Emulsion. Biomacromolecules 2017, 18, 266–271. 10.1021/acs.biomac.6b01615. 27958712

[ref31] Zoppe J. O.; Venditti R. A.; Rojas O. J. Pickering Emulsions Stabilized by Cellulose Nanocrystals Grafted With Thermo-Responsive Polymer Brushes. J. Colloid Interface Sci. 2012, 369, 202–209. 10.1016/j.jcis.2011.12.011. 22204973

[ref32] Tang J. T.; Lee M. F. X.; Zhang W.; Zhao B. X.; Berry R. M.; Tam K. C. Dual Responsive Pickering Emulsion Stabilized by Poly 2-(dimethylamino)ethyl methacrylate Grafted Cellulose Nanocrystals. Biomacromolecules 2014, 15 (8), 3052–3060. 10.1021/bm500663w. 24983405

[ref33] Chen Q. H.; Zheng J.; Xu Y. T.; Yin S. W.; Liu F.; Tang C. H. Surface Modification Improves Fabrication of Pickering High Internal Phase Emulsions Stabilized by Cellulose Nanocrystals. Food Hydrocolloids 2018, 75, 125–130. 10.1016/j.foodhyd.2017.09.005.

[ref34] Hu Z.; Ballinger S.; Pelton R.; Cranston E. D. Surfactant-Enhanced Cellulose Nanocrystal Pickering Emulsions. J. Colloid Interface Sci. 2015, 439, 139–148. 10.1016/j.jcis.2014.10.034. 25463186

[ref35] Nigmatullin R.; Harniman R.; Gabrielli V.; Muñoz-García J. C.; Khimyak Y. Z.; Angulo J.; Eichhorn S. J. Mechanically Robust Gels Formed from Hydrophobized Cellulose Nanocrystals. ACS Appl. Mater. Interfaces 2018, 10 (23), 19318–19322. 10.1021/acsami.8b05067. 29790733

[ref36] Nigmatullin R.; Johns M. A.; Muñoz-García J. C.; Gabrielli V.; Schmitt J.; Angulo J.; Khimyak Y. Z.; Scott J. L.; Edler K. J.; Eichhorn S. J. Hydrophobization of Cellulose Nanocrystals for Aqueous Colloidal Suspensions and Gels. Biomacromolecules 2020, 21 (5), 1812–1823. 10.1021/acs.biomac.9b01721. 31984728

[ref37] Juita; Dlugogorski B. Z.; Kennedy E. M.; Mackie J. C. Low Temperature Oxidation of Linseed Oil: A Review. Fire Science Reviews 2012, 1 (1), 3 10.1186/2193-0414-1-3.

[ref38] Li J.; Feng Q.; Cui J.; Yuan Q.; Qiu H.; Gao S.; Yang J. Self-Assembled Graphene Oxide Microcapsules in Pickering Emulsions for Self-Healing Waterborne Polyurethane Coatings. Compos. Sci. Technol. 2017, 151, 282–290. 10.1016/j.compscitech.2017.07.031.

[ref39] Siva T.; Sathiyanarayanan S. Self Healing Coatings Containing Dual Active Agent Loaded Urea Formaldehyde (UF) Microcapsules. Prog. Org. Coat. 2015, 82, 57–67. 10.1016/j.porgcoat.2015.01.010.

[ref40] Suryanarayana C.; Rao K. C.; Kumar D. Preparation and Characterization of Microcapsules Containing Linseed Oil and Its Use in Self-Healing Coatings. Prog. Org. Coat. 2008, 63 (1), 72–78. 10.1016/j.porgcoat.2008.04.008.

[ref41] Hatami Boura S.; Peikari M.; Ashrafi A.; Samadzadeh M. Self-Healing Ability and Adhesion Strength of Capsule Embedded Coatings-Micro and Nano Sized Capsules Containing Linseed Oil. Prog. Org. Coat. 2012, 75 (4), 292–300. 10.1016/j.porgcoat.2012.08.006.

[ref42] Thanawala K.; Mutneja N.; Khanna A. S.; Raman R. K. S. Development of Self-Healing Coatings Based on Linseed Oil as Autonomous Repairing Agent for Corrosion Resistance. Materials 2014, 7 (11), 7324–7338. 10.3390/ma7117324. 28788249 PMC5510056

[ref43] Hasanzadeh M.; Shahidi M.; Kazemipour M. Application of EIS and EN Techniques to Investigate the Self-Healing Ability of Coatings Based on Microcapsules Filled With Linseed Oil and CeO2 Nanoparticles. Prog. Org. Coat. 2015, 80, 106–119. 10.1016/j.porgcoat.2014.12.002.

[ref44] Szabo T.; Telegdi J.; Nyikos L. Linseed Oil-Filled Microcapsules Containing Drier and Corrosion Inhibitor - Their effects on self-healing capability of paints. Prog. Org. Coat. 2015, 84, 136–142. 10.1016/j.porgcoat.2015.02.020.

[ref45] Shahidi Zandi M.; Hasanzadeh M. The Self-Healing Evaluation of Microcapsule-Based Epoxy Coatings Applied on AA6061 Al Alloy in 3.5% NaCl Solution. Anti-Corros. Methods Mater. 2017, 64 (2), 225–232. 10.1108/ACMM-01-2016-1640.

[ref46] Kim D. M.; Song I. H.; Choi J. Y.; Jin S. W.; Nam K. N.; Chung C. M. Self-Healing Coatings Based on Linseed-Oil-Loaded Microcapsules for Protection of Cementitious Materials. Coatings 2018, 8 (11), 404 10.3390/coatings8110404.

[ref47] Abdipour H.; Rezaei M.; Abbasi F. Synthesis and Characterization of High Durable Linseed Oil-Urea Formaldehyde Micro/Nanocapsules and Their Self-Healing Behaviour in Epoxy Coating. Prog. Org. Coat. 2018, 124, 200–212. 10.1016/j.porgcoat.2018.08.019.

[ref48] Wang H. R.; Zhou Q. X. Evaluation and Failure Analysis of Linseed Oil Encapsulated Self-Healing Anticorrosive Coating. Prog. Org. Coat. 2018, 118, 108–115. 10.1016/j.porgcoat.2018.01.024.

[ref49] Fan Q. C.; Lin B. C.; Nie Y.; Sun Q.; Wang W. X.; Bai L. J.; Chen H.; Yang L. X.; Yang H. W.; Wei D. L. Nanocomposite Hydrogels Enhanced by Cellulose Nanocrystal-Stabilized Pickering Emulsions with Self-Healing Performance in Subzero Environment. Cellulose 2021, 28 (14), 9241–9252. 10.1007/s10570-021-04120-1.

[ref50] Jones R. A. L. Soft Condensed Matter; Oxford University Press: 2002.

[ref51] Mikula R. J.; Munoz V. A. Characterization of Emulsions and Suspensions in the Petroleum Industry Using Cryo-SEM and CLSM. Colloids Surf., A 2000, 174 (1–2), 23–36. 10.1016/S0927-7757(00)00518-5.

[ref52] Costa C.; Rosa P.; Filipe A.; Medronho B.; Romano A.; Liberman L.; Talmon Y.; Norgren M. Cellulose-Stabilized Oil-in-Water Emulsions: Structural Features, Microrheology, and Stability. Carbohydr. Polym. 2021, 252, 117092 10.1016/j.carbpol.2020.117092. 33183583

