# Supplementary material for: Octylamine-Modified Cellulose Nanocrystal-Enhanced Stabilization of Pickering Emulsions for Self-Healing Composite Coatings
Source: ACS Appl Mater Interfaces. 2022 Mar 7;14(10):12722–33. doi: 10.1021/acsami.2c01324 (PMC9096801; doi:10.1021/acsami.2c01324)
Supplement: Supplementary file 1 — am2c01324_si_001.pdf [file am2c01324_si_001.pdf]

Supplementary Information

Octylamine Modified Cellulose Nanocrystal Enhanced Stabilization of Pickering Emulsions for Self-Healing Composite Coatings

Guofan Xu[1], *Rinat Nigmatullin*[1], *Todor T. Koev*[2], *Yaroslav Z. Khimyak*[2], *Ian. P. Bond*[1], *Stephen J. Eichhorn*[1*]

1. Bristol Composites Institute, CAME School of Engineering, University of Bristol, University Walk, Bristol, BS8 1TR, UK.

2. School of Pharmacy, University of East Anglia, Norwich Research Park, NR4 7TJ.

Corresponding Authors

Stephen J. Eichhorn (E-mail: s.j.eichhorn@bristol.ac.uk)

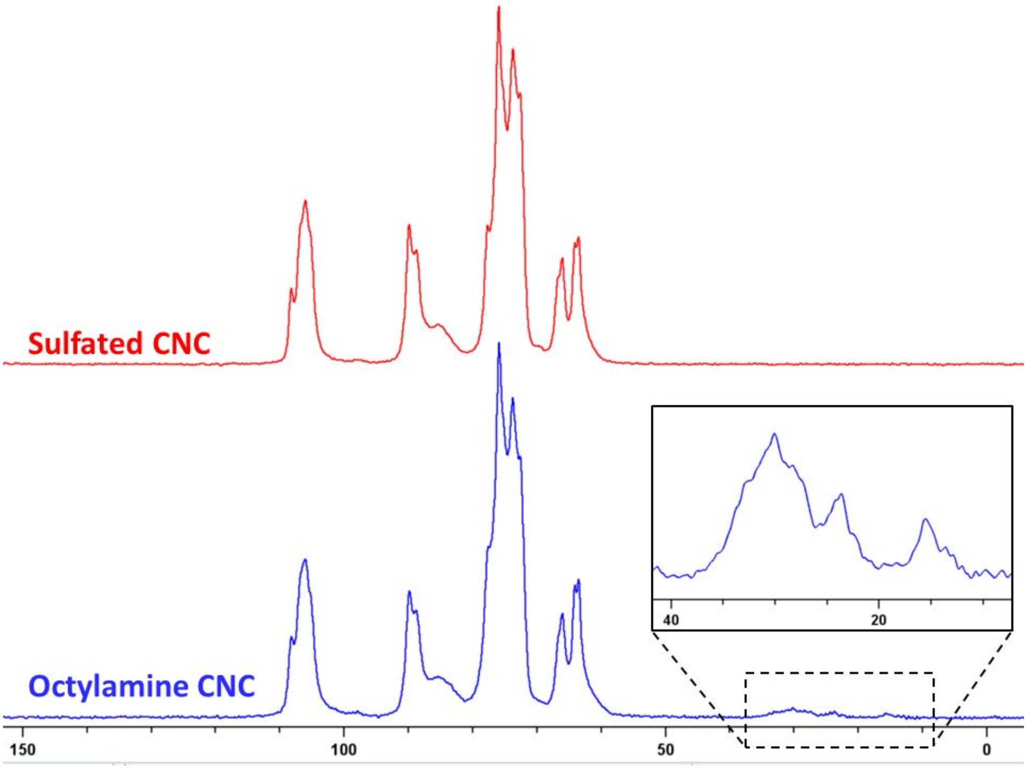

Figure S1. ^{1}H-^{13}C CP/MAS NMR spectral overlay of oCNC (blue) and sulfated CNC (red). Inset shows a zoomed-in spectrum of the octyl moiety in oCNC (*ca.* 12-36 ppm.)

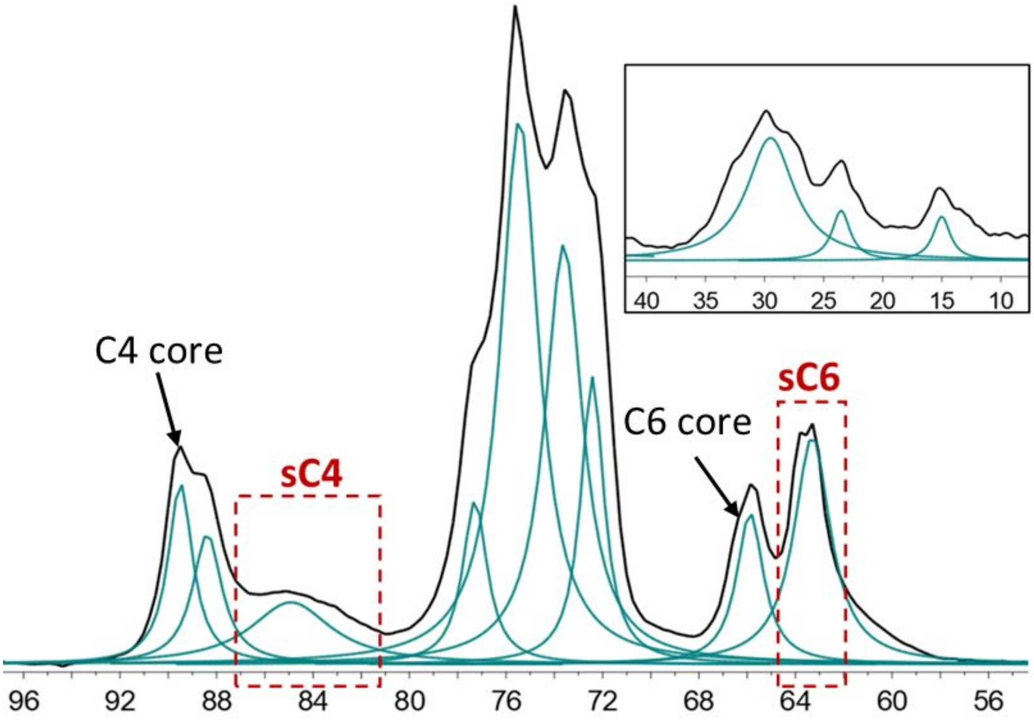

Figure S2: ^{1}H-^{13}C CP/MAS spectral deconvolution of oCNC. Red dashed lines annotate C4 and C6 spectral regions associated with oCNC surface moieties (sC4 and sC6, respectively). Inset shows deconvolution of the octyl moiety in oCNC.

Similar to the NMR methods used by Nigmatullin *et al.*[1, 2] , the surface carbon 4 or 6 and the octylamine moieties were detected by ^{1}H-^{13}C CP-MAS spectra. It was shown in a previous study that ^{1}H-^{13}C CP-MAS spectra of cellulose can be considered as quantitative for CP contact times larger than 600 μs.[3] Carbons 4 and 6 in CNC present distinct chemical environments for the core (C4 and C6 core) and surface (sC4 and sC6) domains of the nanocrystals (Figure S2).[4] This allows discrimination of the surface and bulk domains from the areas of the C4 or C6 surface peak and the C4 or C6 core peaks.

The peak areas were obtained from deconvolution of the CP spectrum in TopSpin 3.5 (Figure S2). Furthermore, integration of carbon peaks of the octyl group (corresponding to 8 carbon atoms) allowed the derivation of the degree of surface functionalization (DSF) according to Eq. S1

$$DSF = \frac{A_{octyl}}{8 \cdot A_S} \qquad \text{Eq. S1}$$

where A_{octyl} is the sum of deconvoluted areas of all octyl peaks and A_{S} the area of the C4 or C6 surface peak. The factor 8 accounts for the number of carbons of the octyl moiety.[1]

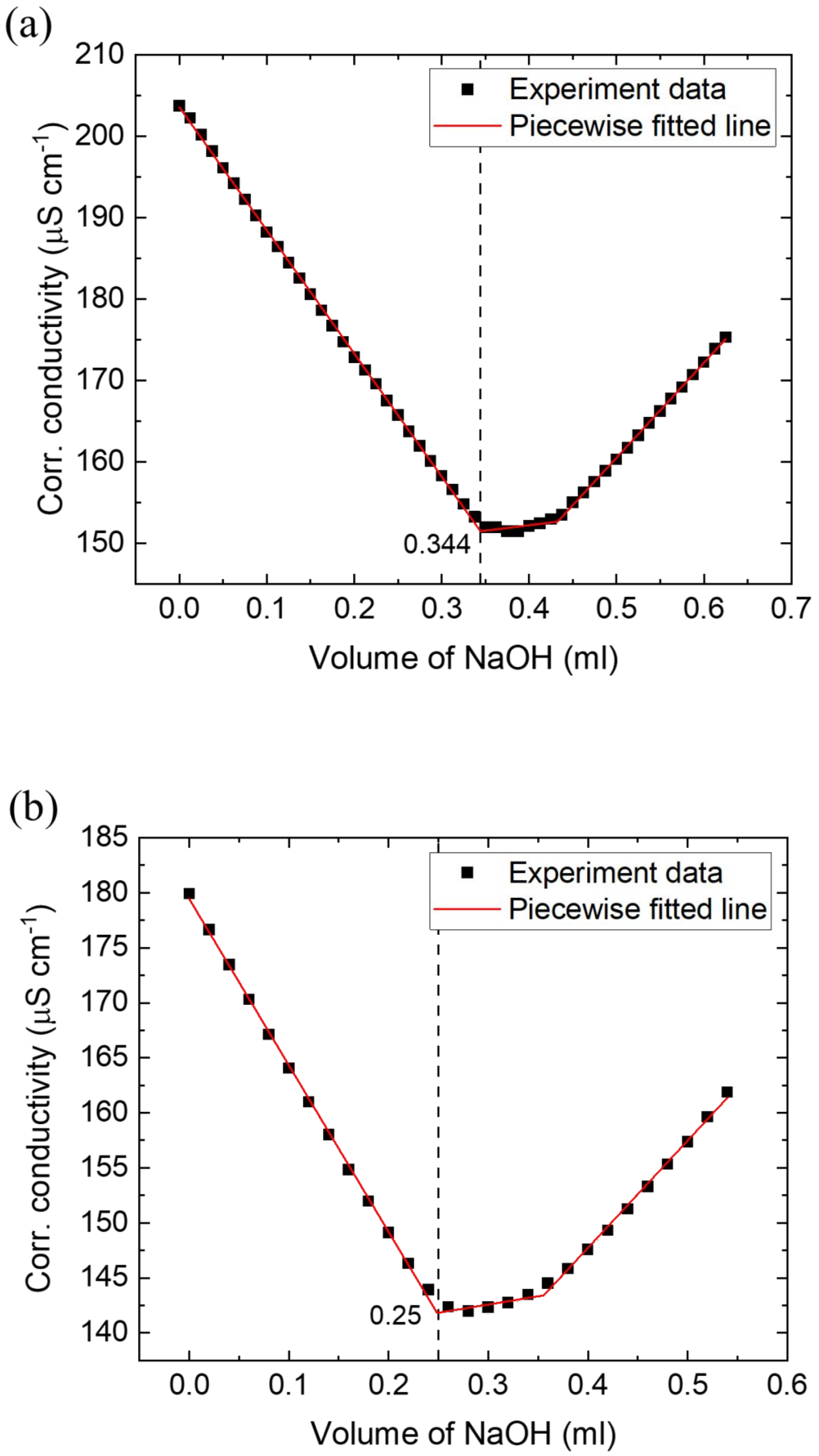

Figure S3. Typical graphs of conductivity titration of (a) freeze-dried sCNCs and (b) oCNCs .

The average surface charge densities of cellulose nanocrystals (CNCs) with sulfate half ester charge groups (both sCNCs and oCNCs) were measured through conductivity titration after purification and changing the counter ions from sodium to protons.[5, 6] For freeze-dried sodium-form sCNCs, the samples were re-dispersed in water with 0.5 wt% concentration and dialyzed against DI water for 3 days to remove any free sodium ions. The suspension was then poured into a column filled with Dowex Marathon C hydrogen form strong acid cation (SAC) exchange resin and a fritted glass disk at the bottom. The sodium ions on the sCNC surfaces are exchanged to protons when the suspension passes through the SAC resin[5]. The resulted acid-form sCNC suspension was then titrated with NaOH to get the mean sulfate half-ester content on the CNC surface. oCNCs were also protonated with SAC resin before titration with the same method. The equivalent points in Figure S1 were determined by fitting the experimental data points into piecewise lines with Chi-Square tolerance value of 1×10^{-9}. The first equivalent points were recorded for surface charge calculation in each test and triplicate tests were performed for each CNC sample. The content of $-OSO_3H$ was 172.22 ± 5.09 mmol kg-1 for the oCNC samples and 245.17 ± 10.43 mmol kg-1 for sCNC samples.

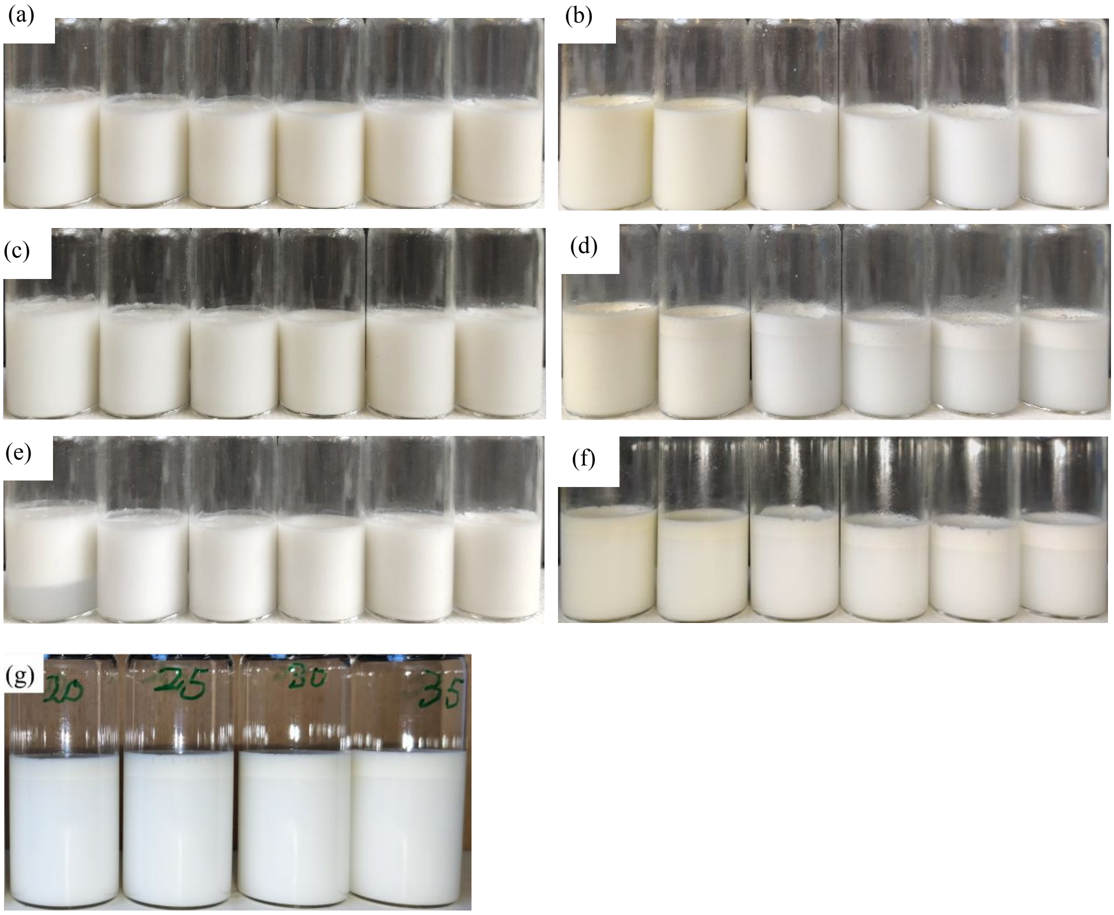

Figure S4. Linseed oil in water emulsions stored at room temperature (a) 10%~35% octyl-CNC stabilized emulsions right after sonication (b) 10%~35% sCNC stabilized emulsions right after sonication (c) 10%~35% octyl-CNC stabilized emulsions 1 hour after sonication (d) 10%~35% sCNC stabilized emulsions 1 hour after sonication (e) 10%~35% octyl-CNC stabilized emulsions 24 hours after sonication (f) 10%~35% sCNC stabilized emulsions 24 hours after sonication (g) 20%~35% sodium dodecyl sulfate stabilised emulsions 10 minutes after sonication.

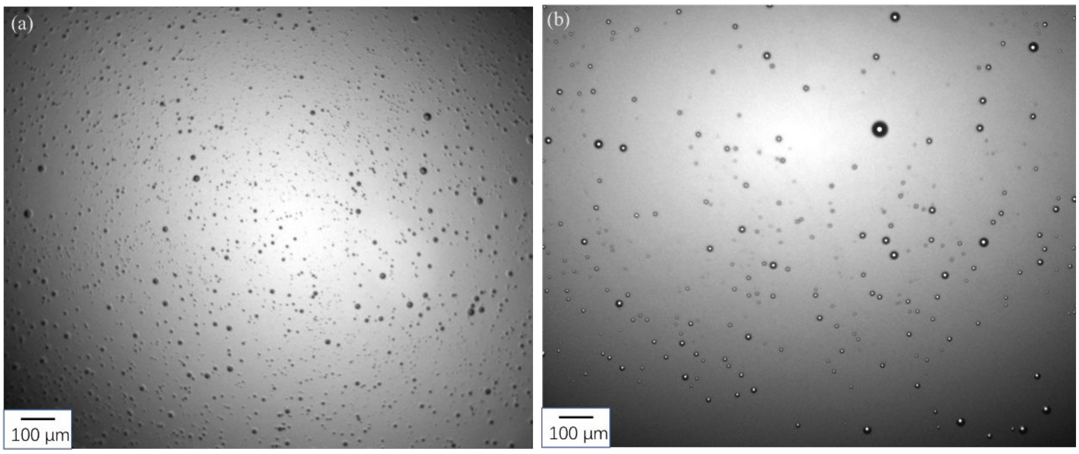

Figure S5. Optical microscope of separated water phases below the creaming layers of (a) 10% oCNC emulsion (b) 10% sCNC emulsion.

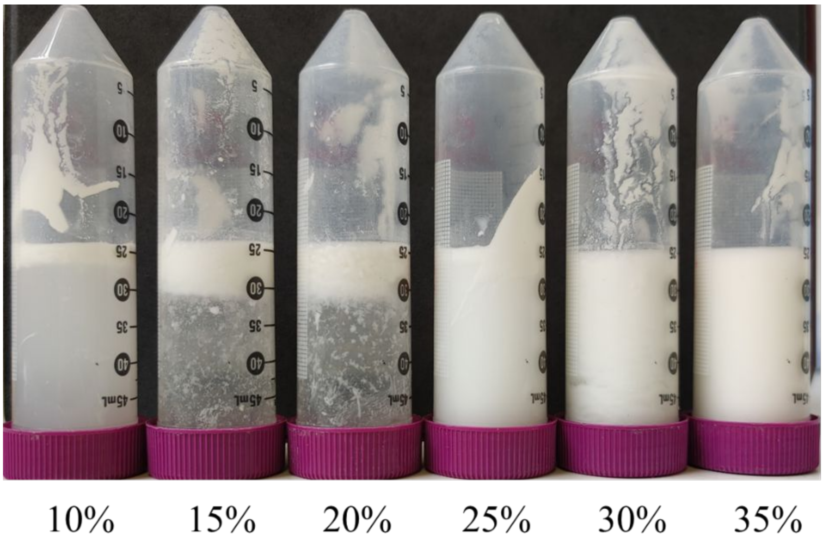

Figure S6. 10%~35% OCNC stabilized emulsions after centrifugation.

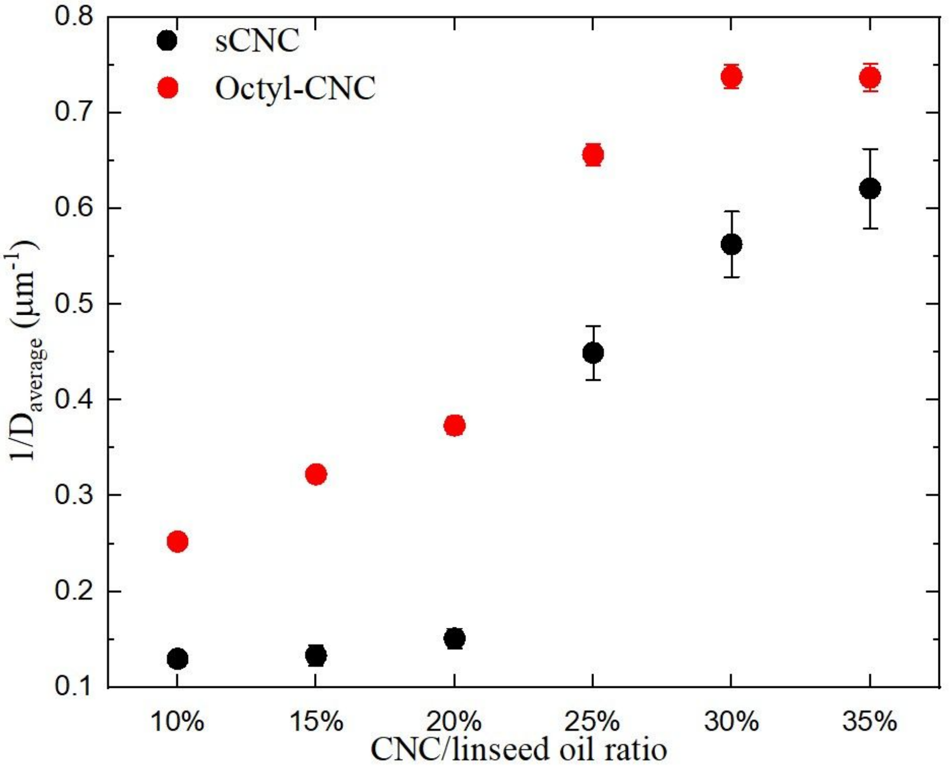

Figure S7. Reciprocal of droplets average diameter as a function of the CNC/oil ratio. Error bars are standard errors from the mean

References

1. Nigmatullin, R.; Harniman, R.; Gabrielli, V.; Muñoz-García, J. C.; Khimyak, Y. Z.; Angulo, J.; Eichhorn, S. J., Mechanically Robust Gels Formed from Hydrophobized Cellulose Nanocrystals. *ACS Appl. Mater. Interfaces* **2018,** *10* (23), 19318-19322.
2. Nigmatullin, R.; Johns, M. A.; Muñoz-García, J. C.; Gabrielli, V.; Schmitt, J.; Angulo, J.; Khimyak, Y. Z.; Scott, J. L.; Edler, K. J.; Eichhorn, S. J., Hydrophobization of Cellulose Nanocrystals for Aqueous Colloidal Suspensions and Gels. *Biomacromolecules* **2020,** *21* (5), 1812-1823.
3. Larsson, P. T.; Wickholm, K.; Iversen, T., A CP/MAS13C NMR Investigation of Molecular Ordering in Celluloses. *Carbohydrate Research* **1997,** *302* (1), 19-25.
4. Brinkmann, A.; Chen, M.; Couillard, M.; Jakubek, Z. J.; Leng, T.; Johnston, L. J., Correlating Cellulose Nanocrystal Particle Size and Surface Area. *Langmuir* **2016,** *32* (24), 6105-6114.
5. Beck, S.; Méthot, M.; Bouchard, J., General Procedure for Determining Cellulose Nanocrystal Sulfate Half-Ester Content by Conductometric Titration. *Cellulose* **2015,** *22* (1), 101-116.
6. Foster, E. J.; Moon, R. J.; Agarwal, U. P.; Bortner, M. J.; Bras, J.; Camarero-Espinosa, S.; Chan, K. J.; Clift, M. J. D.; Cranston, E. D.; Eichhorn, S. J.; Fox, D. M.; Hamad, W. Y.; Heux, L.; Jean, B.; Korey, M.; Nieh, W.; Ong, K. J.; Reid, M. S.; Renneckar, S.; Roberts, R.; Shatkin, J. A.; Simonsen, J.; Stinson-Bagby, K.; Wanasekara, N.; Youngblood, J., Current Characterization Methods For Cellulose Nanomaterials. *Chem. Soc. Rev.* **2018,** *47* (8), 2609-2679.
